# Eugenol and lidocaine inhibit voltage-gated Na^+^ channels from dorsal root ganglion neurons with different mechanisms

**DOI:** 10.3389/fphar.2024.1354737

**Published:** 2024-06-26

**Authors:** Luiz Moreira-Junior, Jose Henrique Leal-Cardoso, Antonio Carlos Cassola, Joao Luis Carvalho-de-Souza

**Affiliations:** ^1^ Department of Anesthesiology, University of Arizona, Tucson, AZ, United States; ^2^ Superior Institute of Biomedical Sciences, State University of Ceará, Fortaleza, Brazil; ^3^ Department of Physiology and Biophysics, Biomedical Sciences Institute, University of Sao Paulo, São Paulo, Brazil

**Keywords:** eugenol, lidocaine, voltage-gated sodium channels, inhibition, state-dependent interactions, dorsal root ganglion neurons, patch-clamp technique

## Abstract

Eugenol (EUG) is a bioactive monoterpenoid used as an analgesic, preservative, and flavoring agent. Our new data show EUG as a voltage-gated Na^+^ channel (VGSC) inhibitor, comparable but not identical to lidocaine (LID). EUG inhibits both total and only TTX-R voltage-activated Na^+^ currents (I_Na_) recorded from VGSCs naturally expressed on dorsal root ganglion sensory neurons in rats. Inhibition is quick, fully reversible, and dose-dependent. Our biophysical and pharmacological analyses showed that EUG and LID inhibit VGSCs with different mechanisms. EUG inhibits VGSCs with a dose–response relationship characterized by a Hill coefficient of 2, while this parameter for the inhibition by LID is 1. Furthermore, in a different way from LID, EUG modified the voltage dependence of both the VGSC activation and inactivation processes and the recovery from fast inactivated states and the entry to slow inactivated states. In addition, we suggest that EUG, but not LID, interacts with VGSC pre-open–closed states, according to our data.

## Introduction

Phytochemicals are a good source of molecules to drive the discovery of new bioactive compounds. Terpenes, molecules formed by isoprene molecule condensation, are the largest class of phytochemicals, with over 25,000 molecules identified (Harrewijn et al., 2012; Kozioł et al., 2014). Cyclic monoterpenoids, found as components of the aromatic essential oil of many plants, are cycled and oxygenated molecules based on two isoprene unit monoterpenes (Clarke and Clarke, 2008). Cyclic monoterpenoids possess a broad range of biological activities, both *in vitro* and *in vivo*. These activities include analgesic (Jorkjend and Skoglund, 1990; Ohkubo and Shibata, 1997; Park et al., 2011; Guimarães et al., 2013), anti-arrhythmogenic (Sensch et al., 2000; Magyar et al., 2004; Tomasova et al., 2015; Binu et al., 2017), antiepileptogenic (Müller et al., 2006; Jeong et al., 2015; Sucher and Carles, 2015), anticonvulsant (Dallmeier and Carlini, 1981; Dallmeier et al., 1983; de Almeida et al., 2011; Nóbrega et al., 2014; Sancheti et al., 2014), and myorelaxant effects (Beer et al., 2007; Lima et al., 2011; Olivoto et al., 2014; Peixoto-Neves et al., 2014; Peixoto-Neves et al., 2015), and they were corroborated by mechanisms of action studies strongly suggesting that the effects mentioned above, at least in part, are caused by interactions between these cyclic monoterpenoid molecules and ion channels expressed in the membranes of excitable cells. Vast literature studies show that cyclic monoterpenoids block and/or modulate voltage-gated sodium channels (VGSCs) (Haeseler et al., 2002; Park et al., 2006; Cho et al., 2008; Park et al., 2009; Moreira-Lobo et al., 2010; Gaudioso et al., 2012; Joca et al., 2012; Silva-Alves et al., 2013; Wang et al., 2015; Teixeira-Fonseca et al., 2021), voltage-gated calcium channels (Magyar et al., 2004; Lee et al., 2005; Soares et al., 2007; Chung et al., 2008; Seo et al., 2013), and voltage-gated potassium channels (Sensch et al., 2000; Magyar et al., 2002; dos Santos-Nascimento et al., 2015). In addition, somatosensory-related transient receptor potential cation channel subfamily A member 1 (TRPA1) (Karashima et al., 2007; Lee et al., 2008; Chung et al., 2014; Takaishi et al., 2014) and other channels are also reported to be modulated by cyclic monoterpenoids (Hall et al., 2004; Li et al., 2008; Yeon et al., 2011; Lee et al., 2015).

VGSCs are membrane proteins comprising an ion-conductive and voltage-sensitive alpha subunit and a varying number of regulatory beta subunits (Hille, 2001; Brackenbury and Isom, 2011; Bouza and Isom, 2018). Most of the known VGSC blockers and inhibitors are directed to the alpha subunit since it possesses the voltage-sensing mechanism and the coupled Na^+^-permeable conductive pore. In mammalian organisms, there are nine different VGSC alpha subunits that share more than 75% identity. Expressed in the cell membrane, each VGSC alpha subunit contains more than 2,000 amino acids and 24 membrane-spanning segments organized in 4 homologous domains radially disposed for a Na^+^-selective conducting pore in the center. In each domain, transmembrane segments S1–S4 form the voltage sensor that provides voltage dependence to the open probability of the central pore that is formed by the S5 and S6 segments from all domains (Pan et al., 2018). VGSCs are the most important ion channels for cell excitability since they are responsible for initiating action potentials in neurons and muscle cells (Hille, 2001; Catterall, 2012). VGSCs are crucial to maintaining physiologic cell excitability in neurons, myocytes, and endocrine cells. Natural variants of VGSCs are major causes of diseases such as epilepsy, periodic paralysis, arrhythmias, and pain disorders (Hodgkin and Huxley, 1952; Hille, 2001; George, 2005; Catterall, 2017; Jiang et al., 2022; Hernandez and Richards, 2023).

Therefore, the development of new blockers and inhibitors for VGSCs could potentially lead to the discovery of new therapies for many diseases of cell excitability (Theile and Cummins, 2011; Meng et al., 2014; Bagal et al., 2015). Currently, there is a need for VGSC inhibitors or modulators that would target the right subtype of these channels for tissue and cell specificity.

The present work aimed at speculating a putative state-dependent inhibition of VGSCs expressed by cultured dorsal root ganglion (DRG) neurons by eugenol (4-allyl-2-methoxyphenol, EUG), a bioactive cyclic monoterpenoid that is traditionally used as a dentistry material in humans in dental temporary dressings, where it provides analgesic and anti-inflammatory activities (Hume, 1939; Hume, 1984).

Our data show that EUG reversibly inhibits voltage-activated sodium currents (I_Na_) in a dose-dependent manner without altering membrane resistance. Next, we investigated a possible state-dependent inhibition of VGSCs by EUG. To this end, we investigated the inhibition of the total I_Na_ and of tetrodotoxin-resistant (TTX-R) I_Na_ separately. TTX is one of the few partially specific modulators of VGSCs since it intensively blocks seven of the nine different VGSCs. Therefore, TTX provides a strategy to study different VGSCs when they are expressed in somatic cells. We studied total or TTX-R I_Na_ only, in response to EUG, and we compared the results with the inhibition and modulation induced by lidocaine (LID), a classical local anesthetic that inhibits VGSCs. Our data suggest that EUG may interact with the pre-open–closed states of the channels to enhance the inhibition of total I_Na_, in addition to the inhibition of VGSCs in their resting states, which is remarkably different from the effects induced by LID.

Additionally, we looked into a possible inhibiting interaction between EUG and LID, when inhibiting VGSCs, in an attempt to speculate about the unknown EUG VGSC-inhibiting mechanisms. The inhibitory mechanisms of EUG and LID are similar but not the same, suggesting hypothetically different inhibition mechanisms on different VGSCs that are impossible to determine with studies like the one presented here. The currents we used here to study these drugs are mediated by many types of VGSCs that are expressed in DRG neurons.

We propose EUG as a new structural scaffold for the development of new target-specific VGSC modulators. The new suggestive inhibiting mechanism, based on the notion of the interaction between EUG and the pre-open–closed states of VGSCs that we have possibly identified in our studies as attributable to EUG, has not been described before for a cyclic monoterpenoid such as EUG, and it is currently a matter of further studies by our group.

## Results

### EUG quickly inhibits I_Na_, and the inhibition is fully reversible

DRG neurons express a variety of VGSCs. Despite lacking a specific population of channels for new inhibitor studies, these cells provide an outstanding platform to study VGSCs natively expressed in their physiological environment. Additional benefits of studying VGSCs in DRG neurons are the practicality of cell preparation, the cell sizes ranging from 20 to 50 μm, and the absence of dendrites that may result in space clamp issues when these structures are present. We used voltage-gated K^+^ channel blockers, voltage gated-Ca^2+^ channel blockers, and online leak subtraction to isolate the voltage-activated I_Na_ for our experiments (see *Methods*).

We tested the fast inhibition by EUG on I_Na_ expressed in DRG neurons and compared the results with the inhibition produced by LID, a local anesthetic used in clinics that inhibits VGSCs to produce anesthesia. In a separate batch of experiments, we used 300 nM of tetrodotoxin (TTX) during the whole experiment to inhibit only the TTX-sensitive part of the total I_Na_ in DRG neurons (Roy and Narahashi, 1992; Elliott and Elliott, 1993; Ogata and Tatebayashi, 1993; Tan et al., 2014). This procedure enables the recording of only the TTX-R fraction of I_Na_ that is important in the peripheral nervous system as it relates to sensorial information such as the ones produced by noxious stimuli (Djouhri et al., 2003; Gudes et al., 2015; Bennett et al., 2019).

**FIGURE 1:**
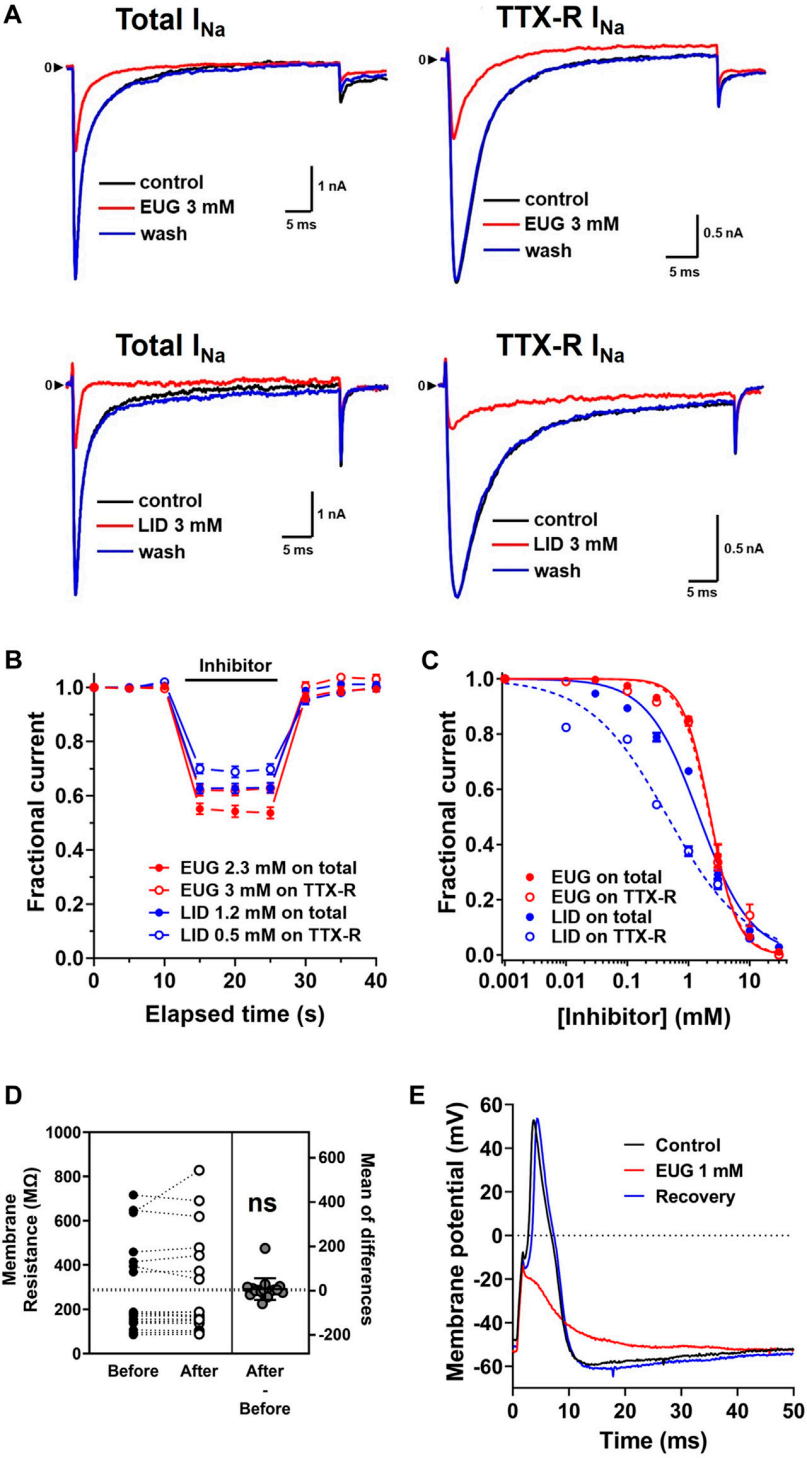
EUG inhibits the total I_Na_ and TTX-R I_Na_ in a dose-dependent and fully reversible manner. **(A)** Representative traces showing the inhibition of total I_Na_ and only TTX-R I_Na_ activated at +20-mV membrane potential, from a holding potential of −110 mV, by EUG and LID for comparison. **(B)** Fast inhibition and full recovery by EUG or LID on the peak of total I_Na_ or only TTX-R I_Na_, followed by full recovery upon drug washout in a depolarization time series of 0.2 Hz. **(C)** Averaged fractional currents (symbols) were plotted for dose–response curves. Vertical bars denote SEM (n > 6 per point). Continuous lines are the best fit using Eq. 1 (see *Methods* for details). Filled symbols are from the total I_Na_, and empty symbols are from TTX-R I_Na_. The datasets plotted in red relate to EUG, and those in blue relate to LID. EUG inhibits total I_Na_ and TTX-R I_Na_ with IC_50_ of 2.27 ± 0.07 mM (n = 30) and 2.21 ± 0.08 mM (n = 32), respectively. For comparison, LID inhibits the total I_Na_ and TTX-R I_Na_ with IC_50_ values of 1.42 ± 0.17 mM (n = 24) and 0.44 ± 0.09 mM (n = 26), respectively. **(D)** Paired data show that 3 mM EUG does not significantly alter the membrane resistance of the cells. The mean of differences is shown, and it is not significantly different from zero (two-tailed paired *t*-test, n = 15; ns: not significant). **(E)** Typical action potential waveform recorded from a random DRG neuron upon a 1-ms 1-nA depolarizing current injection. EUG at a concentration of 1 mM promptly inhibits action potential firing, and the effect is quickly removed upon drug washout.

EUG and LID quickly inhibit the total I_Na_ and TTX-R I_Na_ in a concentration-dependent manner, and the inhibition is fully reversible after drug washout (Figure 1A, B). By using an in-house fast single-cell superfusion system, we show that EUG inhibits I_Na_ in less than 5 s upon its addition to the experiments. The inhibition may be even faster than 5 s, but we used a time series of 0.2 Hz to avoid inhibition accumulation during faster series. In addition, we used a holding potential of −110 mV in all our experiments in an effort to remove VGSCs from their inactivated states, to maximize the currents we recorded.

**FIGURE 2:**
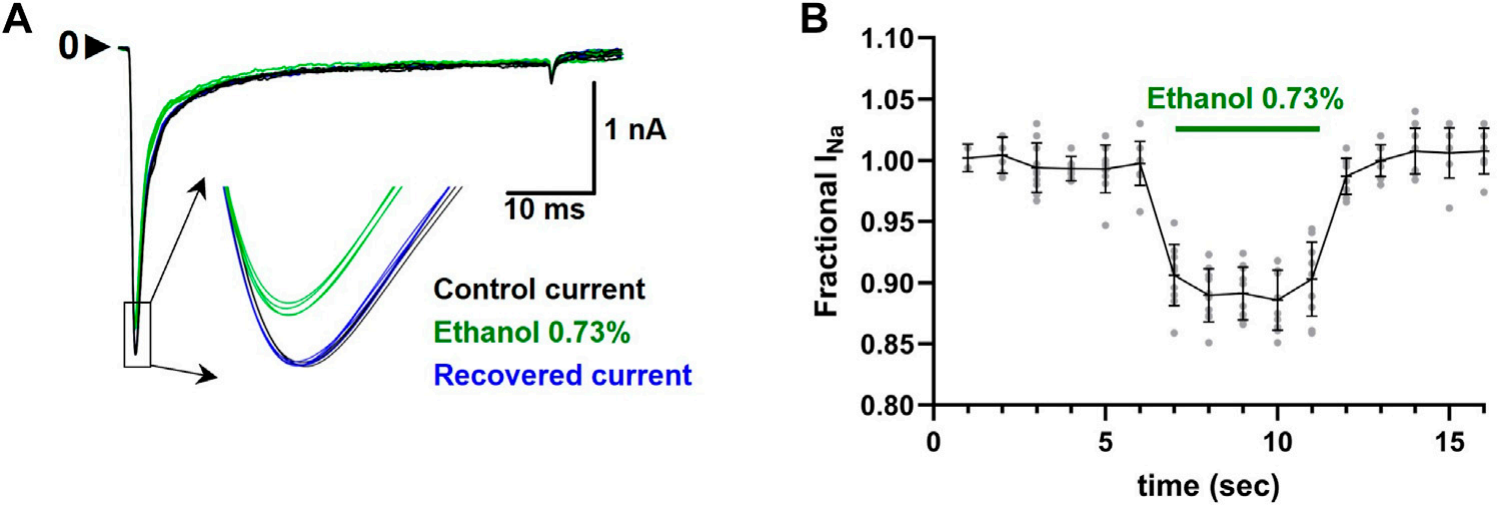
Effect of ethanol 0.73% (vol/vol) on the total I_Na_ peaks. **(A)** Representative traces showing the inhibition of the current peaks (inset) by ethanol 0.73% (green traces). Recovered currents are also shown in blue. **(B)** Average ± SEM (n = 10) I_Na_ peak inhibition by ethanol 0.73% is shown as black lines, and individual values are shown as gray circles.

EUG was solubilized with ethanol and then diluted in experimental (external) solution to the desired concentration. The maximal final concentration of ethanol was 0.73% (vol/vol), and this amount was used to solubilize EUG for 10 and 30 mM working solutions only. For lower final concentrations of EUG, we used a molar solubilization ratio of 1:21 (EUG:ethanol). At 0.73% (vol/vol), ethanol reversibly blocks 10% of the total I_Na_ peak (Figure 2). For reference, the concentration of ethanol used to solubilize EUG at 2 mM was 0.2%.

EUG inhibits the total I_Na_ with an IC_50_ value of 2.27 ± 0.07 mM (n = 30) and TTX-R I_Na_ with an IC_50_ value of 2.21 ± 0.08 mM (n = 32). Using an identical approach, we found that LID inhibited the total I_Na_ with an IC_50_ value of 1.42 ± 0.17 mM (n = 24) and TTX-R I_Na_ with an IC_50_ value of 0.44 ± 0.09 mM (n = 26) (Figure 1C). All IC_50_ values for the inhibition of I_Na_ and TTX-R I_Na_ by EUG and LID are shown in Table 1. It is noteworthy to mention that none of the EUG concentrations utilized in the present study affected the membrane resistance of the neurons. As an example, we show paired data with absolute membrane resistance values before and after 3 mM EUG is added to the experiment (Figure 1D). For these experiments, we used a hyperpolarizing pulse to −130 mV from the usual −110 mV holding potential. In addition, we tested EUG on action potential firing in a representative neuron. As predicted, 1 mM EUG prevents neurons from firing action potentials, a neuronal capability fully recovered a few seconds after EUG washout (Figure 1E).

**TABLE 1:** Dose–response parameters (IC_50_ and Hill coefficients) for the inhibition of total and TTX-R I_Na_ by EUG, LID, and combinations as indicated.

		IC_50_ (mM)	Hill coefficient
Total I_Na_	EUG	2.27 ± 0.075^#^, ****	1.95 ± 0.120^#^, ****
	LID	1.42 ± 0.169	1.02 ± 0.169
	EUG (LID 0.5 mM)	1.84 ± 0.274†, **	0.98 ± 0.140†, ****
	LID (EUG 1.3 mM)	0.73 ± 0.126^#,^ ****	1.02 ± 0.167
TTX-R I_Na_	EUG	2.21 ± 0.080^#^, ****	1.84 ± 0.115^#^, ****
	LID	0.44 ± 0.087	0.67 ± 0.088

Dose–response curves were built with n > 6 cells for each drug concentration separately, and the data were fitted with Eq. 1 (see *Methods*). Extra sum-of-squares F-test comparing the fit parameter with that of LID alone (^#^) and with EUG alone (†). **p < 0.01, ***p < 0.001, and ****p < 0.0001.

Remarkably, the inhibition profile of the total I_Na_ by EUG differs from that of LID in the slope of the dose-dependent inhibition curves, the Hill coefficient (EUG slope = 1.95 ± 0.120 vs. LID slope = 1.02 ± 0.169; extra sum-of-squares F-test *p* < 0.0001), suggesting that at least part of the inhibiting mechanism is different between these two drugs.

### EUG inhibits total I_Na_ and TTX-R I_Na_ at all activating membrane potentials

**FIGURE 3:**
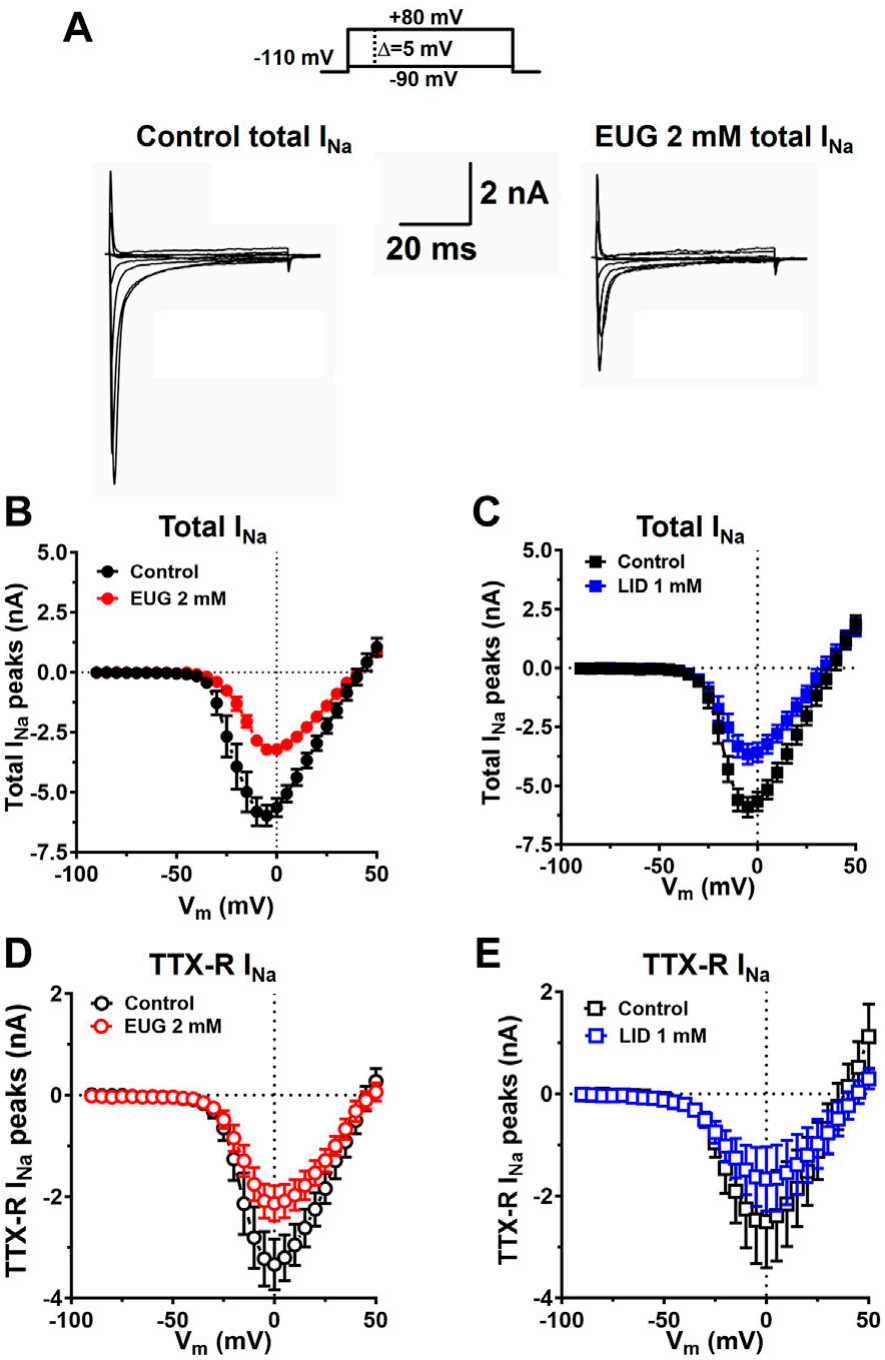
EUG inhibits I_Na_ at any depolarized membrane potential stimulus. **(A)** Typical families of the total I_Na_ under control conditions (absence of EUG) and in the presence of EUG at 2 mM. **(B–E)** Current-to-voltage (I-V) relationships for the peaks of the total I_Na_, and only TTX-R I_Na_ are shown in the absence and presence of EUG at a concentration of 2 mM or LID at a concentration of 1 mM, as indicated. Average I-V relationships are shown as symbols, and vertical bars denote SEM (n > 9). Filled symbols are from the total I_Na_, and empty symbols are from TTX-R I_Na_, as indicated. The datasets plotted in red relate to EUG, and those in blue relate to LID.

From the holding potential of −110 mV, we applied depolarizing voltage steps from −90 mV to +50 mV in increments of +5 mV every 5 s (0.2 Hz). EUG at a concentration of 2 mM inhibits the total I_Na_ and TTX-R I_Na_, as activated by all voltages without significantly affecting the reversal potential of the currents. Comparable results were found when LID at a concentration of 1 mM was tested as an inhibitor using identical procedures (Figure 3).

**FIGURE 4:**
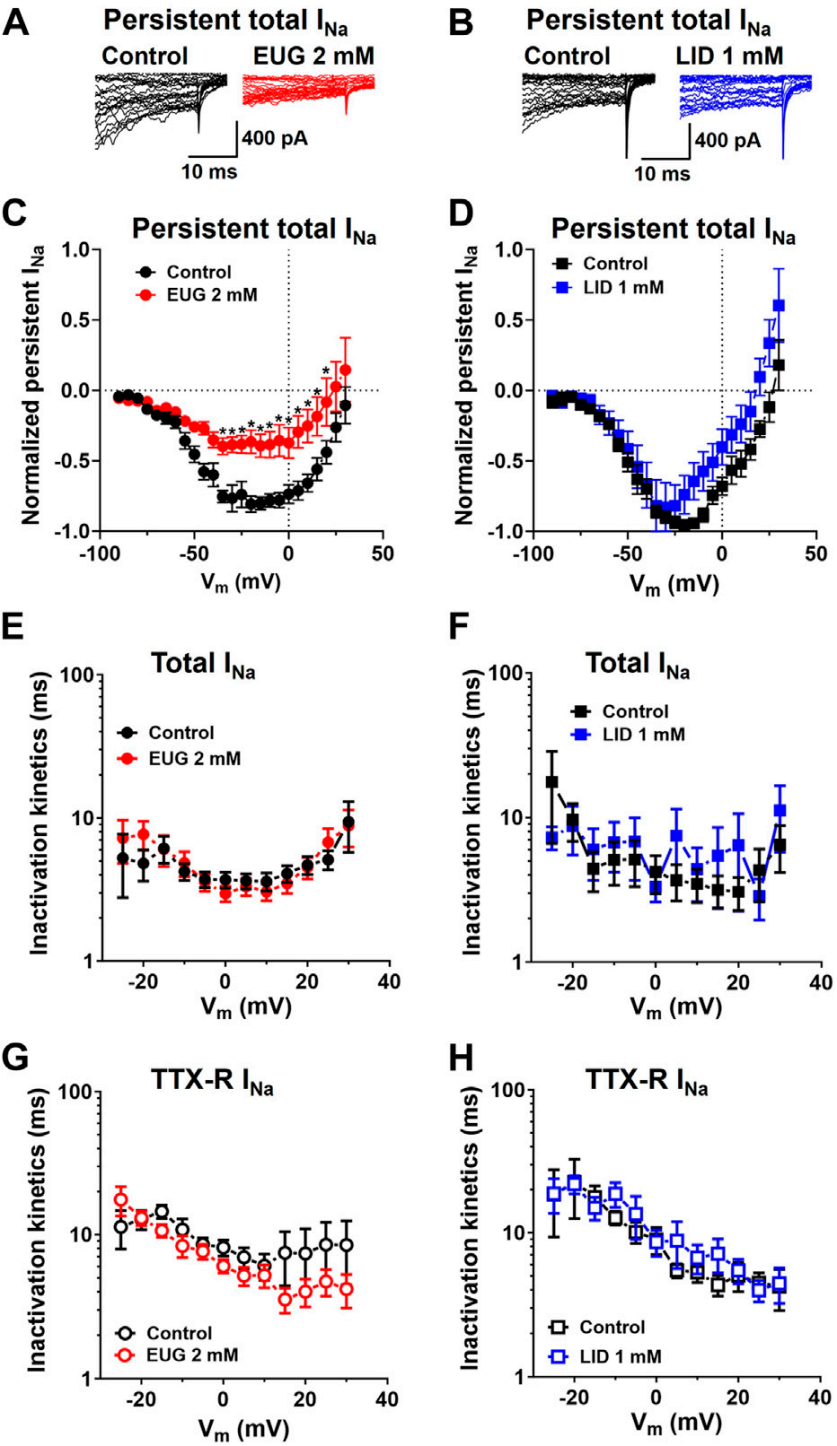
EUG inhibits persistent total I_Na_ without affecting the time constant of I_Na_ inactivation. **(A,B)** Typical total I_Na_ traces showing the last 30 ms of 50-ms depolarizing pulses before (control) and after EUG or LID is added to the experiment at the indicated concentrations. Note the persistent total I_Na_ at the end of the depolarizing pulses right before the tail currents. **(C,D)** Typical I-V relationships of persistent total I_Na_ recorded at the end of 50-ms depolarizing pulses. EUG effectively inhibits persistent total I_Na_ (two-way ANOVA and Šídák’s multiple comparisons test; *: *p* < 0.05). Note that despite the consistent inhibition of the total I_Na_ peaks (Figure 3C), LID fails to inhibit persistent Na^+^ currents. Average persistent I_Na_ I-V relationships are shown as symbols, and vertical bars denote SEM (*n* > 9). **(E–H)** Total I_Na_ and TTX-R I_Na_ inactivation time constants kinetics are unaffected by EUG or LID. Inactivation time constants were taken by a double exponential fit (Eq. 2) of the decaying phase of the I_Na_ during a wide range of different depolarizing pulses, as indicated in the graphs. Average weighted inactivation time constants (Eq. 3) at different membrane potentials are shown as symbols, and vertical bars denote SEM (*n* > 9). Filled symbols are from the total I_Na_, and empty symbols are from TTX-R I_Na_, as indicated. The datasets plotted in red relate to EUG, and those in blue relate to LID.

Interestingly enough, the persistent total I_Na_ after 50-ms depolarization was remarkably inhibited by EUG but not by LID (Figures 4A–D). Nevertheless, neither EUG nor LID changed the kinetics of the inactivation process of total I_Na_ or TTX-R I_Na_ during a test pulse (Figures 4E–H), as discussed later.

### EUG shifts the voltage dependence of Na^+^ conductance activation to more depolarized membrane potentials

**FIGURE 5:**
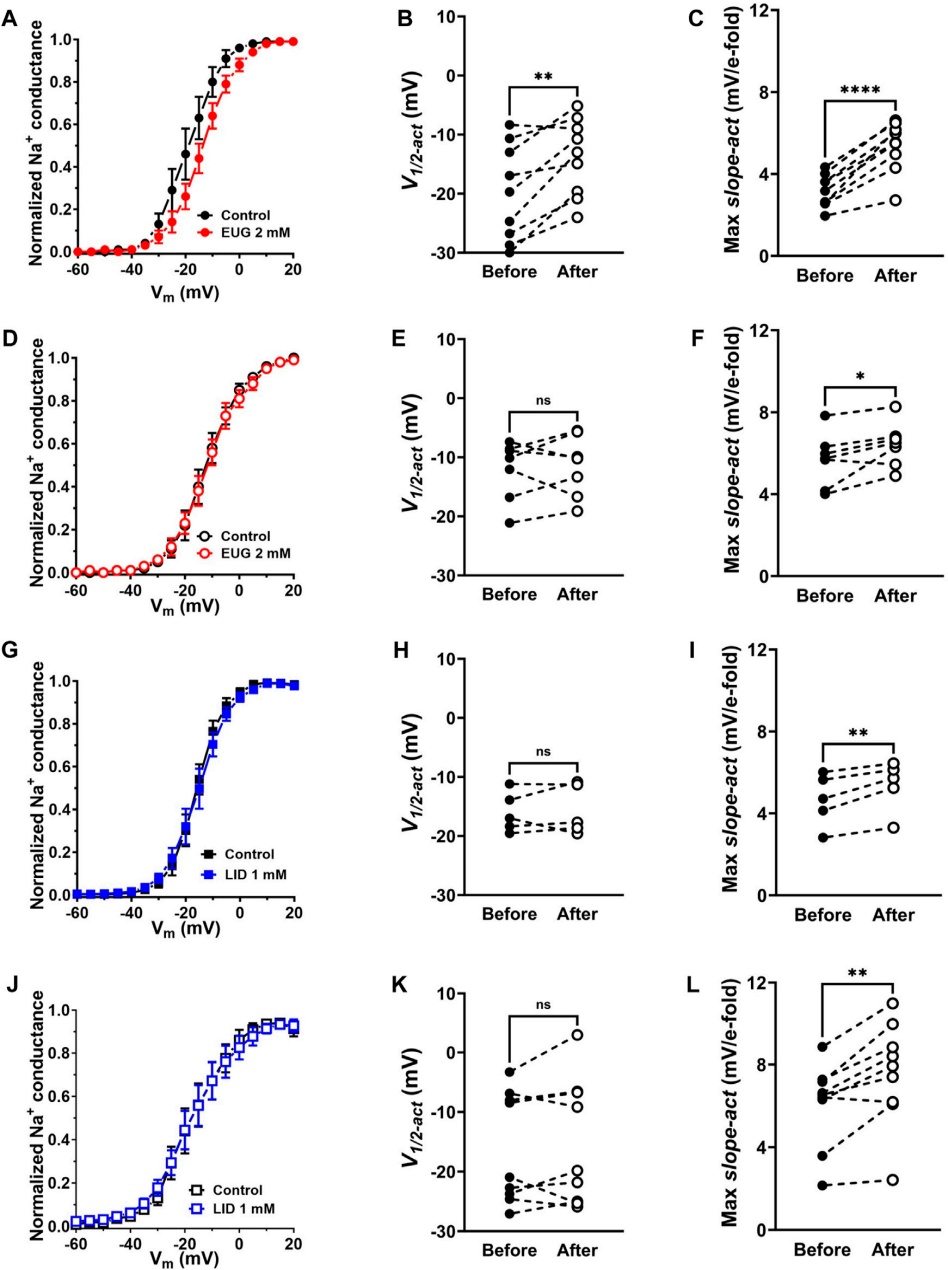
EUG changes the voltage dependence of total I_Na_ activation. **(A–C)** Normalized and averaged Na^+^ conductance–voltage (G-V) curves in the absence (control) and presence of EUG at a concentration of 2 mM. Data from individual cells were plotted as individual G-V curves and fitted with Eq. 5 (See *Methods*) for voltage dependence of the I_Na_ activation (*V*_*1/2-act*_) **(B)** and voltage sensitivity (*Max slope-act*) **(C)** parameters, before and after EUG is added. Averaged G-V curves are shown as symbols, with vertical bars denoting SEM (n > 9). **(D–F)** EUG did not shift the voltage dependence of TTX-R I_Na_ **(E)** but decreased its voltage sensitivity **(F)**. **(G–L)** LID also did not shift the voltage dependence of either total **(H)** or TTX-R I_Na_ **(K)**, but it decreased the voltage sensitivity of both currents [**(I,L)**, respectively]. Filled symbols are from total I_Na_, and empty symbols are from TTX-R I_Na_, as indicated. The datasets plotted in red relate to EUG, and those in blue relate to LID. **(B,C,E,F,H,I,K,L)** Note that the statistically significant difference, at different levels, between conditions before and after EUG or LID is added, is indicated by asterisks (two-tailed paired *t*-test; ns: not significant; *p < 0.05; **p < 0.01; and ****p < 0.0001).

We transformed I_Na_ peak values from each separate cell into Na^+^ conductance by using Ohm’s law (Eq. 4; see *Methods*) before normalizing all values by their maxima. We then plotted these cell-specific data against the activating membrane potential to produce conductance–voltage (G-V) curves in the absence and presence of 2 mM EUG or 1 mM LID for comparison. We also averaged data to highlight voltage-dependence shifts when they existed (Figures 5A, D, G, J). To the individual cell normalized Na^+^ conductance curves, we fitted Boltzmann’s equation (inactivation kinetics; Eq. 5; see *Methods*) for the individual voltage dependence of the activation process (*V*_*1/2-act*_) and the voltage sensitivity of the process (max *slope-act*). On average, EUG significantly shifted *V*_*1/2-act*_ of the total I_Na_ but not of the TTX-R I_Na_ to more depolarized membrane potentials, and it decreased the max *slope-act* of both total I_Na_ and TTX-R I_Na_ (Figures 5A–F). Data from the inhibition of I_Na_ by LID show no changes in *V*_*1/2-act*_ of both total I_Na_ and TTX-R I_Na_ and a change in the max *slope-act* of TTX-R I_Na_ only (Figures 5G–L). Figures 5A, D, G, J show the average curves that hide the individual variations in *V*_*1/2-act*_ and max *slope-act*. Averaged fitting parameters and statistical analysis are shown in Table 2.

**TABLE 2:** Voltage activation and inactivation of total and TTX-R I_Na_ in the absence and presence of EUG or LID.

		V_1/2-act_ (mV)	Max slope-act (mV/e-fold)	V_1/2-inact_ (mV)	Max slope-inact (mV/e-fold)
Total I_Na_	Control (EUG)	−19.9 ± 2.67 (n = 9)	3.2 ± 0.26 (n = 9)	−48.5 ± 2.46 (n = 9)	−10.5 ± 0.67 (n = 9)
	EUG 2 mM	−13.8 ± 2.18 (n = 9) **	5.4 ± 0.41 (n = 9) ****	−63.85 ± 2.92 (n = 9) ****	−12.2 ± 10.7 (n = 9) ns
	Control (LID)	−16.0 ± 1.53 (n = 5)	4.7 ± 0.57 (n = 5)	−45.7 ± 2.72 (n = 6)	−10.5 ± 0.67 (n = 6)
	LID 1 mM	−15.6 ± 1.87 (n = 5) ns	5.4 ± 0.55 (n = 5) *	−50.6 ± 3.04 (n = 6) *	−10.2 ± 0.50 (n = 6) ns
TTX-R I_Na_	Control (EUG)	−12.1 ± 1.90 (n = 7)	5.7 ± 0.50 (n = 7)	−31.7 ± 2.36 (n = 7)	−5.67 ± 0.49 (n = 7)
	EUG 2 mM	−11.5 ± 1.96 (n = 7) ns	6.4 ± 0.4 (n = 7) **	−36.9 ± 2.48 (n = 7) **	−5.83 ± 0.43 (n = 7) ns
	Control (LID)	−16.2 ± 3.09 (n = 9)	6.0 ± 0.67 (n = 9)	−28.8 ± 2.09 (n = 9)	−5.7 ± 0.48 (n = 9)
	LID 1 mM	−15.3 ± 3.51 (n = 9) ns	7.6 ± 0.84 (n = 9) **	−32.6 ± 2.86 (n = 9) *	−7.2 ± 1.24 (n = 9) ns

Note: paired t-test: ns, not significant; *p < 0.05; **p < 0.01; ***p < 0.001; and ****p < 0.0001.

### EUG shifts the voltage dependence of total I_Na_ and TTX-R I_Na_ inactivation to more negative membrane potentials

**FIGURE 6:**
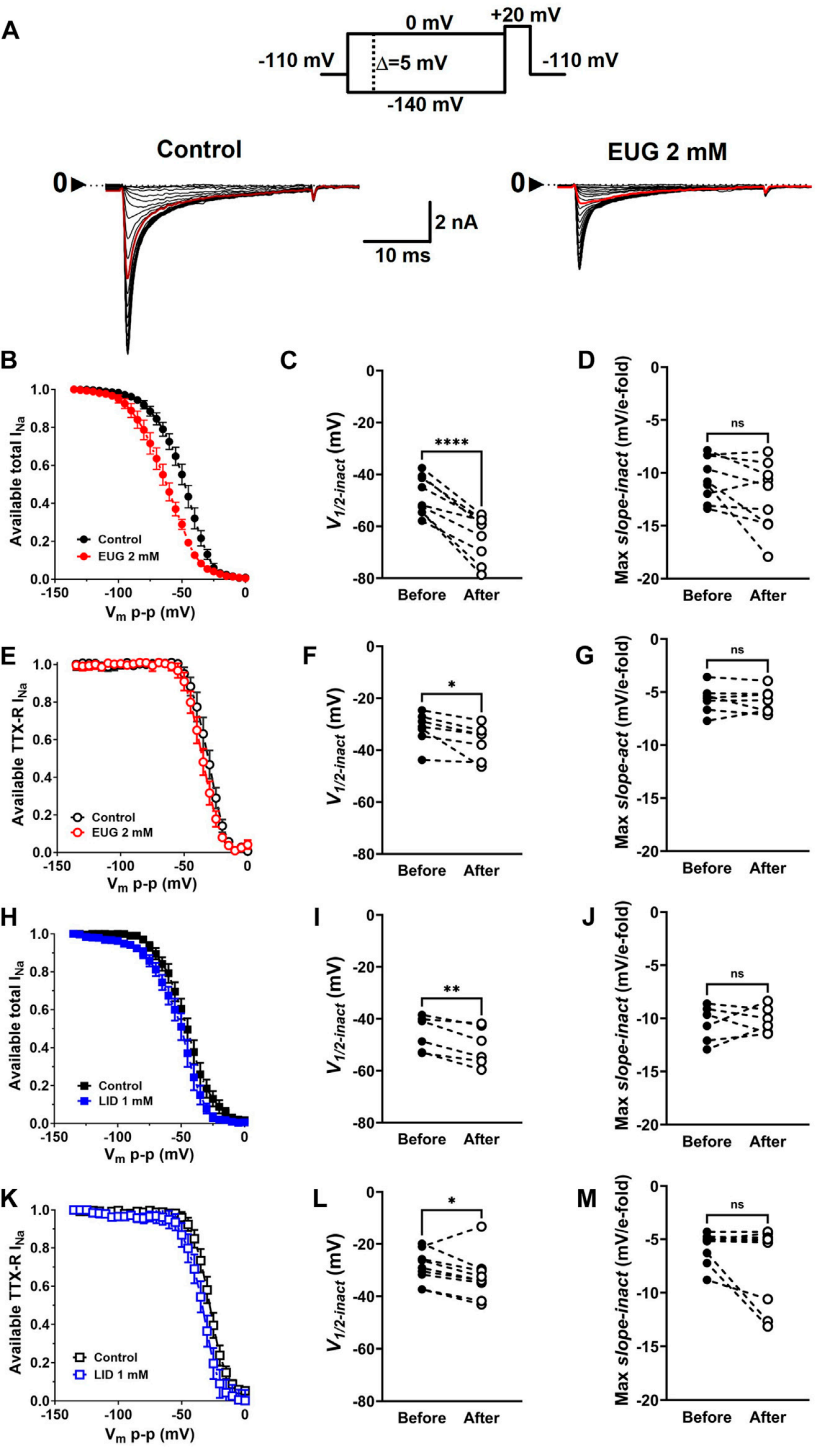
EUG changes the voltage dependence of I_Na_ inactivation. **(A)** Typical families of total I_Na_ recorded at +20 mV after 100-ms conditioning pre-pulses at voltages ranging from −140 to 0 mV (V_m_p-p, upper panel) under control conditions (absence of EUG, left panel) and in the presence of EUG at 2 mM (right panel). In both families of currents, the red trace relates to a V_m_p-p value of −50 mV. **(B,E,H,K)** Normalized and averaged available total I_Na_ or TTX-R I_Na_ in the absence (control) and presence of EUG or LID at indicated concentrations were plotted against V_m_p-p for inactivation curves. Averaged values are shown as symbols, and vertical bars denote SEM (n > 9). Data from individual cells were plotted as individual curves and fitted with Eq. 6 (see *Methods*) for *V*_*1/2-inact*_ (the voltage dependence of the inactivation process) and *Max slope-inact* (the voltage sensitivity of the process). EUG shifts the inactivation curves of both the total I_Na_ **(C)** and TTX-R I_Na_ **(F)** to more negative voltages without altering their voltage sensitivity **(D,G)**. **(H–M)** LID shifted the voltage dependence of both the total **(I)** and TTX-R I_Na_ **(L)** without changing the voltage sensitivity **(J,M)**. All filled symbols relate to the total I_Na_, and the empty symbols relate to TTX-R I_Na_, as indicated. The datasets plotted in red relate to EUG, and those in blue relate to LID. **(C,D,F,G,I,J,L,M)** Note that the statistically significant difference, at different levels, between conditions before and after EUG or LID is added, is indicated by asterisks (two-tailed paired *t*-test; ns: not significant; *p < 0.05; **p < 0.01; and ****p < 0.0001).

In addition to activation, one of the greatest properties of VGSCs is their fast inactivation process. This process limits the action of a VGSC to a few milliseconds, thus preventing long depolarizing periods that would impair the right functioning of cells in most cases. We tested EUG on the inactivation process of I_Na_ by studying the level of inactivation of the total I_Na_ and TTX-R I_Na_ after many depolarized (−140 mV–0 mV at every 5 mV) 100-ms conditioning pre-pulses. The inactivation level was tested by a pulse to +20 mV right after the conditioning pulse (Figure 6A). Graphically, we expressed the normalized and average levels of non-inactivated I_Na_ against the conditioning pre-pulse voltage to build steady-state inactivation curves (Figures 6B, E, H, K). Individual inactivation curves were fitted with Boltzmann’s equation (Eq. 6), similar to what was done with G-V curves for their voltage dependence (*V*_*1/2-inact*_) and voltage sensitivity (max *slope-inact*). EUG enhances the voltage-dependent inactivation of the total I_Na_ and TTX-R I_Na_ by shifting their voltage dependence to more negative voltages. The shift in the curves from the total I_Na_ was highly significant, nearly −15 mV on average. The shift in the curves from TTX-R I_Na_ was also significant and −5 mV on average. LID also shifted the inactivation curves of both the total I_Na_ and TTX-R I_Na_ to more negative potentials but not to the extent that EUG did, with −5-mV and −4-mV shifts on average, respectively. Neither EUG nor LID effectively changed the voltage sensitivity of the fast inactivation process of I_Na_ (Figure 6). Averaged fitting parameters and statistical analysis results for I_Na_ inactivation analysis are shown in Table 2.

### EUG affects the recovery from the inactivation of total I_Na_

**FIGURE 7:**
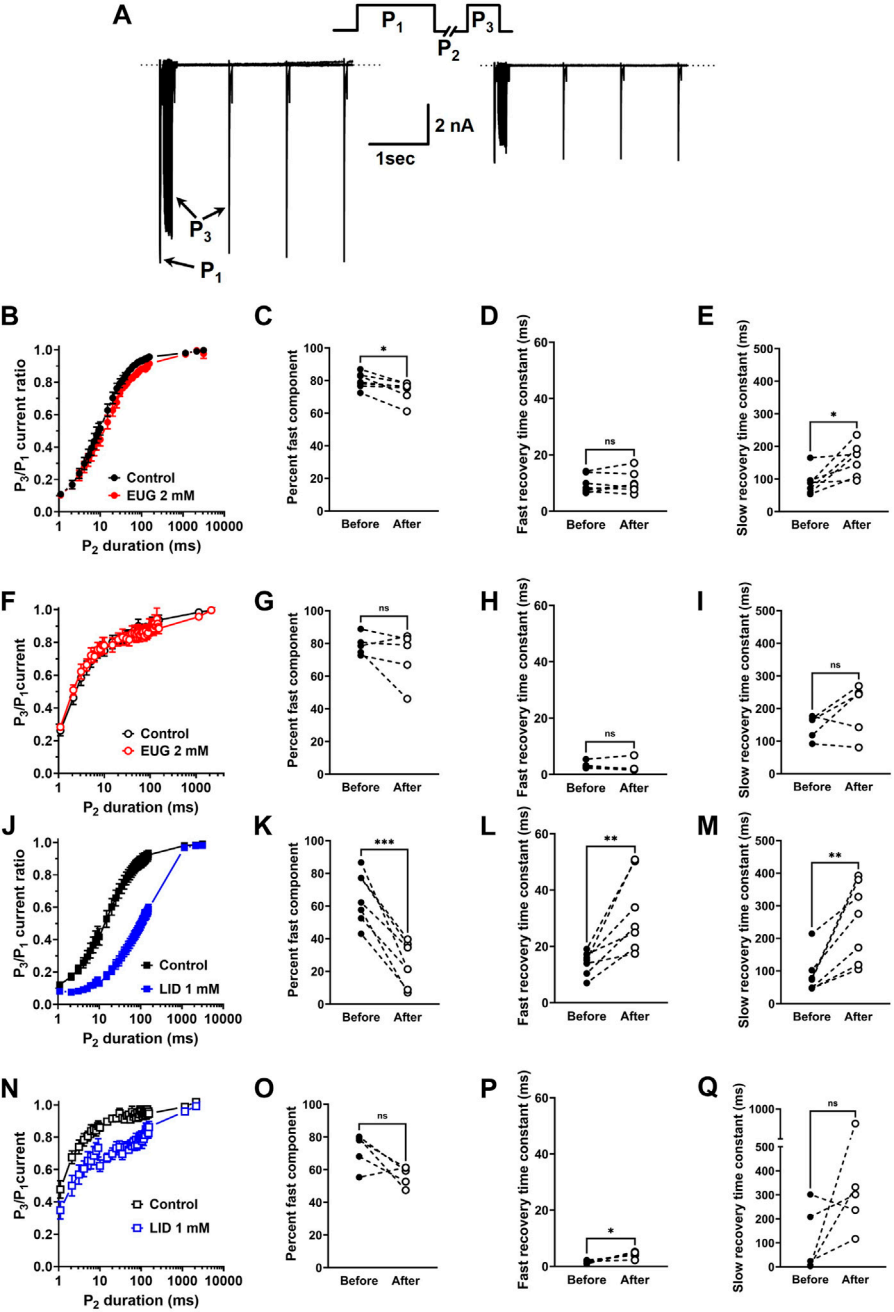
EUG affects I_Na_ recovery from inactivation. **(A)** Typical families of the total I_Na_ recorded with the classic three-pulse voltage-clamp protocol with P_1_, P_2_, and P_3_ are shown in the upper panel. I_Na_ are activated by P_1_ and P_3_ under control conditions (absence of EUG, left panel) and in the presence of EUG at 2 mM (right panel) (see text for details). **(B,F,J,N)** Normalized and averaged P_3_/P_1_ current ratios at different P_2_ durations in the absence (control) and presence of EUG or LID at indicated concentrations are plotted for recovery curves. Data from individual cells were plotted as individual curves and fitted with Eq. 7 for respective percent of fast component and kinetics of fast and slow components (time constants *τ*_fast_ *and τ*_slow_) (see *Methods*). **(C, D, E)** Individual cell parameters in the absence and presence of EUG (before and after, respectively) are shown as percent fast component and fast and slow recovery time constants. **(F–I)** EUG did not affect any recovery from the inactivation parameters of TTX-R I_Na_. Recovery from inactivation data is shown as symbols, and vertical bars denote SEM (n > 9). **(J–Q)** For comparison, similar experiments were performed using LID as an I_Na_ inhibitor. As expected, LID delays recovery from inactivation in both total and TTX-R I_Na_. **(B,F,J,N)** Filled symbols are from the total I_Na_, and empty symbols are from TTX-R I_Na_, as indicated. The datasets plotted in red relate to EUG, and those in blue relate to LID. **(C–E, G–I, K–M, O–Q)** Note that the statistically significant difference, at different levels, between conditions before and after EUG or LID is added, is indicated by asterisks (two-tailed paired *t*-test; ns: not significant; *p < 0.05; **p < 0.01; and **p < 0.001).

We evaluated the speed of the recovery from the fast inactivation of the total I_Na_ and TTX-R I_Na_ in DRG neurons. To do so, we used the classic three-pulse voltage-clamp protocol, which consists of a pulse P_1_ to +20 mV lasting 50 ms to fully fast inactivate I_Na_, a duration-varying pulse P_2_ at a holding potential of −110 mV to recover the currents from inactivation, and a pulse P_3_ to +20 mV once again to activate the fraction of the I_Na_ recovered during P_2_. Typical current recordings show small differences in the kinetics of recovery from inactivation before and after EUG is added to the experiment (Figure 7A). All I_Na_ peaks recorded during P_3_ were normalized by the corresponding (same sweep) I_Na_ peak during P_1_, averaged, and plotted against the duration of P_2_ (Figures 7B, F, J, N). Individual plots from the same cell before and after EUG was added to the experiment were fitted with two exponentials for a fast and slow kinetic component and a proportion amplitude component that we chose to represent as a percent of the fast component (Eq. 7). The statistical analyses of these parameters show that in the experiments with the total I_Na_, but not with TTX-R I_Na_, the percentage of fast component of the recovery from inactivation was significantly decreased from 80% to 74% (Figure 7C). In this same experiment, the slow recovery component of the recovery from inactivation was significantly delayed by EUG (Figures 7B–E). Because most of the recovery of the inactivation process happens at a fast pace (fast component), the delayed slow component did not strongly affect the overall recovery from inactivation. EUG did not affect any parameter of recovery from the inactivation of the TTX-R I_Na_ (Figures 7F–I). For comparison, we ran the same experiments with LID, which is known to delay I_Na_ recovery from inactivation. LID substantially and significantly delays the recovery from the inactivation of both total I_Na_ and TTX-R I_Na_, mainly by affecting the fast component of the process. In the total I_Na_, LID significantly decreases the percent fast component, and it delays both the fast and slow components of the process (Figures 7J–Q). In TTX-R I_Na_, LID acts by significantly delaying the fast component of the process only. Averaged fitting parameters and statistical analysis results for I_Na_ recovery from the inactivation analysis are shown in Table 3.

**TABLE 3:** Recovery from the inactivation of total and TTX-R I_Na_ in the absence and presence of EUG or LID.

		Percent fast component	Fast time constant	Slow time constant
Total I_Na_	Control (EUG)	79.9 ± 1.81 (n = 7)	9.6 ± 1.21 (n = 7)	89.1 ± 14.09 (n = 7)
	EUG 2 mM	73.8 ± 2.30 (n = 7) *	10.2 ± 1.41 (n = 7) ns	161.0 ± 18.6 (n = 7) *
	Control (LID)	65.2 ± 5.91 (n = 7)	14.1 ± 1.56 (n = 7)	87.1 ± 22.60 (n = 7)
	LID 1 mM	24.1 ± 5.00 (n = 7) ***	31.9 ± 5.20 (n = 7) **	252.9 ± 45.77 (n = 7) **
TTX-R I_Na_	Control (EUG)	79.1 ± 2.81 (n = 5)	3.2 ± 0.55 (n = 5)	144.3 ± 16.68 (n = 5)
	EUG 2 mM	71.8 ± 7.11 (n = 5) ns	2.8 ± 1.01 (n = 5) ns	196.7 ± 36.39 (n = 5) ns
	Control (LID)	72.2 ± 4.72 (n = 5)	1.6 ± 0.23 (n = 5)	108.8 ± 61.49 (n = 5)
	LID 1 mM	56.4 ± 2.73 (n = 5) ns	4.1 ± 0.51 (n = 5) *	358.5 ± 117.6 (n = 5) ns

Note: paired t-test: ns, not significant; *p < 0.05; **p < 0.01; ***p < 0.001; ****p < 0.0001.

### EUG-inhibiting effects are not potentiated at high frequencies of depolarization

**FIGURE 8:**
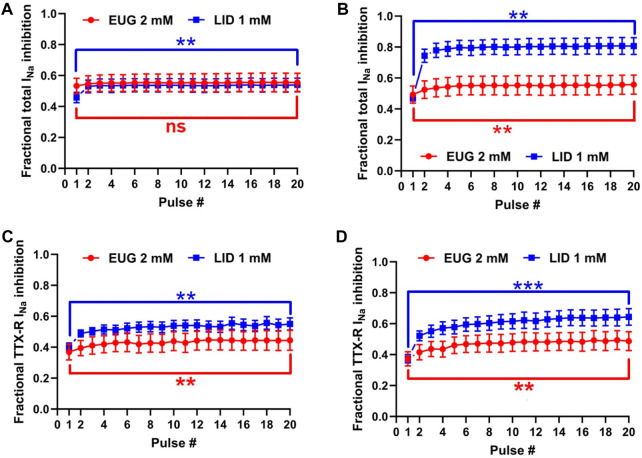
I_Na_ inhibition induced by EUG is weakly potentiated at high-frequency depolarizations. **(A)** Inhibition of total I_Na_ induced by EUG or LID over 20 successive depolarizations to +20 mV at 2 Hz and **(B)** at 5 Hz. It is noteworthy that the first pulse was taken after at least 1 min at a holding potential of −110 mV. **(C, D)** Inhibition of TTX-R I_Na_ induced by EUG or LID in a similar protocol as in **(A,B)**. At 2-Hz stimulation but not at 5-Hz stimulation, EUG performs similar to LID both on total and TTX-R I_Na_. Filled symbols are from total I_Na_, and empty symbols are from TTX-R I_Na_ as indicated. At 5-Hz stimulation, LID performs better than EUG on both the total and TTX-R I_Na_. The datasets plotted in red relate to EUG, and those in blue relate to LID. Note that a statistically significant difference between pulses 1 and 20 in each plot after two-tailed paired t-tests is indicated by asterisks. ns: not significant; **:*p* < 0.01 and ***:*p* < 0.001.

We tested the inhibition intensity of the total I_Na_ and TTX-R I_Na_ by EUG in a time series of depolarizations at 2 and 5 Hz (Figure 8). This test shows the inability of the VGSCs to recover from inactivation in a period between two depolarization events. The voltage clamp protocols used here consisted of 50-ms depolarizing pulses; therefore, they were characterized by a duty cycle of 10% when stimulation was at 2 Hz and 25% when stimulation was at 5 Hz. In other words, at 2 Hz, the I_Na_ represented the recovered currents during a 450-ms period, and at 5 Hz, the recovered currents during a 150-ms period. It is noteworthy that the first pulse in the series was taken after at least 1 min at a holding potential of −110 mV. Typically, 2 mM EUG does not have its fractional inhibition of total I_Na_ significantly increased at 2 Hz, from 0.53 ± 0.049 in the first pulse of the series to 0.55 ± 0.061 in the 20th pulse of the series. At a stimulation of 5 Hz, the fractional inhibition of the total I_Na_ was significantly increased from 0.49 ± 0.056 in the first pulse to 0.55 ± 0.062 in the 20th pulse (two-tailed paired t-tests, *p* = 0.20 for 2 Hz and *p* < 0.01 for 5 Hz). When EUG was used to inhibit TTX-R I_Na_, the fractional inhibition was significantly increased from 0.37 ± 0.051 to 0.44 ± 0.064 at 2 Hz and from 0.37 ± 0.046 to 0.49 ± 0.061 at 5 Hz (two-tailed paired t-tests, *p* < 0.01 for 2 Hz and 5 Hz). For comparison, we also tested LID under the same conditions. At a stimulation of 2 Hz, LID performs similar to EUG both on total and TTX-R I_Na_. With LID as an inhibitor, the total I_Na_ inhibition was significantly increased from 0.46 ± 0.034 to 0.54 ± 0.044 at a stimulation of 2 Hz and from 0.47 ± 0.021 to 0.81 ± 0.055 at 5 Hz (two-tailed paired t-tests, *p* < 0.01 for 2 Hz and 5 Hz). On TTX-R I_Na_, inhibition by LID was significantly potentiated from 0.40 ± 0.025 to 0.55 ± 0.040 at 2 Hz and from 0.37 ± 0.027 to 0.64 ± 0.054 at 5 Hz (two-tailed paired t-tests, *p* < 0.01 for 2 Hz and *p* < 0.001 for 5 Hz). Fractional inhibition data and statistical analysis results for the I_Na_ inhibition series at 2 Hz and 5 Hz are shown in Table 4.

**TABLE 4:** Fractional inhibition of total and TTX-R I_Na_ by EUG or LID at different simulating frequencies.

		2 Hz		5 Hz	
		1st pulse	20th pulse	1st pulse	20th pulse
Total I_Na_	EUG 2 mM	0.53 ± 0.049 (n = 9)	0.55 ± 0.061 (n = 9) ns	0.49 ± 0.056 (n = 8)	0.55 ± 0.062 (n = 8) **
	LID 1 mM	0.46 ± 0.034 (n = 7)	0.54 ± 0.044 (n = 7) **	0.47 ± 0.021 (n = 5)	0.81 ± 0.055 (n = 5) **
TX-R I_Na_	EUG 2 mM	0.37 ± 0.051 (n = 6)	0.44 ± 0.064 (n = 6) **	0.37 ± 0.046 (n = 6)	0.49 ± 0.061 (n = 6) **
	LID 1 mM	0.40 ± 0.025 (n = 8)	0.55 ± 0.040 (n = 8) **	0.37 ± 0.027 (n = 7)	0.64 ± 0.054 (n = 7) ***

Note: Two-tailed paired t-tests: ns: not significant; **p < 0.01; ***p < 0.001.

### EUG does not increase the rate of slow inactivation entry

**FIGURE 9:**
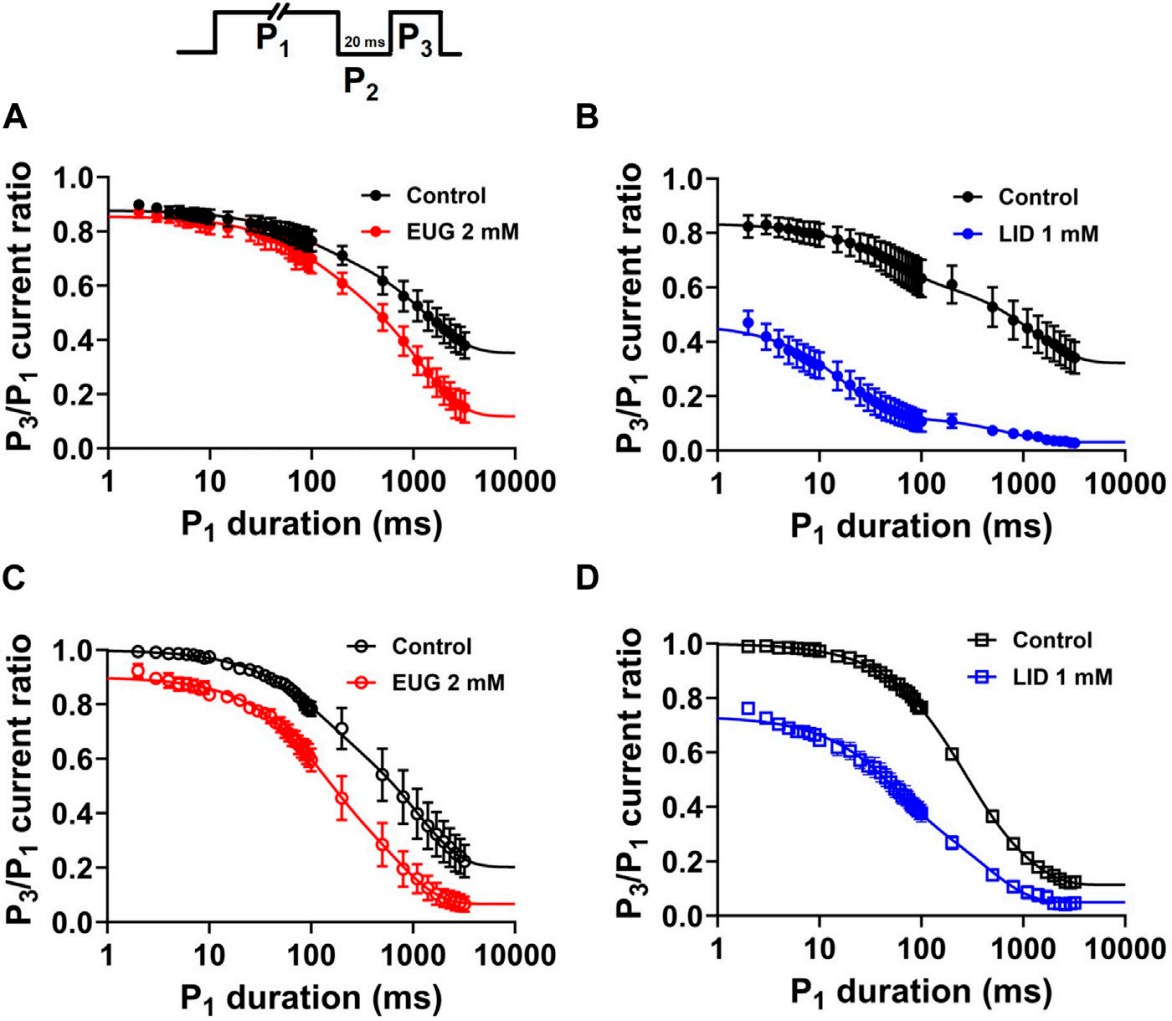
EUG does not affect the slow inactivation state entry. A modified three-pulse voltage-clamp protocol (upper panel) was used to estimate the amount of channels inactivated after a time-varying activating/inactivating P_1_ pulse to +20 mV. A fixed 20-ms P_2_ pulse at holding followed, and finally, a P_3_ pulse to +20 mV again serves to estimate the amount of slow inactivation that occurred during P_1_. **(A,C)** Total I_Na_ and TTX-R I_Na_ represented as the P_3_/P_1_ current fraction were plotted against P_1_ duration in the absence of EUG (control condition) and in the presence of EUG or LID at indicated concentrations. **(B,D)** Data for inhibition with LID are also shown for comparison. EUG does not change the kinetics of the slow inactivation process, and, as expected, LID greatly accelerates slow inactivation kinetics. Filled symbols are from total I_Na_, and empty symbols are from TTX-R INa, as indicated. The data sets plotted in red relate to EUG, and those in blue relate to LID. All plots were fitted with double exponentials (continuous lines; Eq. 8), and the fitting parameters and statistical analysis are shown in Table 5.

VGSCs continue to undergo conformational changes after fast inactivation is completed. After long depolarizations, the channels are in the commonly called slow inactivated states (Vilin and Ruben, 2001). Because of the fast inactivation, slow inactivation cannot be observed directly as current decay like the fast inactivation. We studied slow inactivation with a three-pulse protocol consisting of a duration-varying P_1_ pulse to +20 mV intended to activate and inactivate VGSCs, followed by a 20-ms period at holding P_2_ to recover I_Na_ from fast inactivation, and finally, a P_3_ pulse to +20 mV once again that is intended to activate the available I_Na_ (Figure 9). We plotted averaged P_3_/P_1_ current ratios against the duration of P_1_ to estimate the kinetics of inactivation entry. P_1_ pulses up to 50 ms in duration mostly measure fast inactivation entry. Longer depolarizations drive the channels into slow inactivated states. We used two exponentials (Eq. 8) to fit the data to extract the level of inactivation when P_1_ = 2 ms, as well as the percent of the fast component and the time constants of the P_3_/P_1_ current ratio decay that we use to infer about the slow-inactivation entry kinetics. EUG does not significantly affect any of these two parameters from the total I_Na_ or TTX-R I_Na_. In turn, our data using LID as an inhibitor show it significantly affects both the level of inactivation when P_1_ = 2 ms and the fast and the slow inactivating components of the slow inactivation process. All parameters from the analysis above are summarized in Table 5.

**TABLE 5:** Amplitudes and kinetic parameters of the slow inactivation entry of total I_Na_ and TTX-R I_Na_ in the absence and presence of EUG or LID.

		Fractional current when P_1_ = 2 m	Percent fast component (%)	Fast inactivation entry time constant (ms)	Slow inactivation entry time constant (s)
Total I_Na_	Control (EUG) (*n* = 13)	0.89 ± 0.028	33.40 ± 7.169	741.41 ± 346.032	1829.30 ± 445.187
	EUG 2 mM (n = 12)	0.82 ± 0.034 ^ns^	26.92 ± 4.583 ^ns^	792.48 ± 298.530 ^ns^	1,142.30 ± 246.525 ^ns^
	Control (LID) (n = 14)	0.86 ± 0.028	33.03 ± 4.423	256.83 ± 117.378	1834.31 ± 523.600
	LID 1 mM (n = 15)	0.62 ± 0.067**	59.86 ± 4.320***	25.04 ± 10.996*	292.87 ± 117.879**
TTX-R I_Na_	Control (EUG) (n = 10)	1.00 ± 0.002	31.23 ± 14.283	182.77 ± 103.587	1,601.62 ± 641.124
	EUG 2 mM (n = 8)	0.93 ± 0.028^ns^	47.56 ± 16.257^ns^	76.58 ± 30.563^ns^	631.05 ± 282.320^ns^
	Control (LID) (n = 9)	0.98 ± 0.028	60.00 ± 8.451	236.71 ± 32.997	1,090.23 ± 314.020
	LID 1 mM (n = 9)	0.76 ± 0.049**	35.03 ± 6.913	33.05 ± 11.57****	287.01 ± 49.765*

Note: paired t-test: ns, not significant; *p < 0.05; **p < 0.01; ***p < 0.001; ****p < 0.0001.

### EUG may interact with pre-open states of VGSCs

**FIGURE 10:**
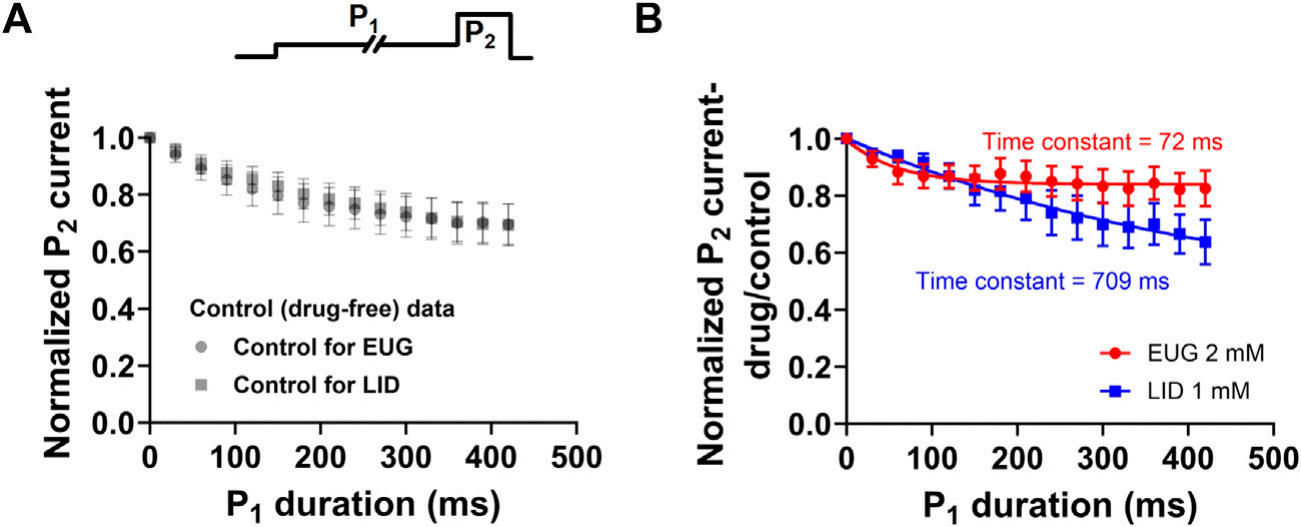
EUG may interact with pre-open–closed states of VGSCs. A time-varying conditioning pulse P_1_ to −70 mV was applied before a I_Na_ activating pulse P_2_ to +20 mV was applied to fully activate available currents, as shown in the upper panel. **(A)** In the absence of drugs, the −70-mV conditioning period (P_1_) decreases the available current activated by P_2_ in a time-dependent manner. The control (drug-free) data in both cases are not statistically different (multiple Student’s t-tests, *p* > 0.05). **(B)** Normalized P_2_ current in the presence of the drug (EUG or LID) relative to control (drug-free) values was plotted against the P_1_ duration. The inhibiting effect of EUG during P_2_ was rapidly increased as P_1_ lasted longer. We fitted a single exponential decay to individual cell data, and the average time constant of the inhibiting effect increase was 72.80 ± 14.926 ms (*n* = 7). For comparison, LID was used instead as an inhibitor, and, despite the inhibition strength increasing with the duration of P_1_, the time course of the process is much slower, and it has a time constant of 709.10 ± 307.018 ms (*n* = 6). The kinetics of EUG inhibition potentiation by P_1_ was statistically different from that of LID (Student’s t-test, *p* = 0.04).

Our data showed that EUG significantly and consistently shifts the total I_Na_ activation curves to more depolarized potentials (Figure 5). In addition, we showed that EUG shifts the steady-state inactivation curve to more negative potentials (Figure 6), although without inducing remarkable changes in the recovery from fast inactivation (Figure 7) and on the use-dependent inhibition that is a hallmark for local anesthetics like LID (Figure 8). One of our hypotheses that would explain the abovementioned data is that EUG interacts with and stabilizes pre-open–closed states of VGSCs (Sunami and Hiraoka, 1996). These pre-open–closed states are short-lived non-conducting states of the channels that are, therefore, not trivial to study (Armstrong, 2006; Lenaeus et al., 2017; Xiao et al., 2021; Gamal El-Din and Lenaeus, 2022). We designed a protocol, which, to the best of our knowledge, has not been used before exactly as is to study the possible interaction between EUG and the pre-open states of VGSCs. The protocol consists of depolarizing the membrane of the neurons from a −110-mV holding potential to −70 mV for various different durations (0–420 m) in a time series. The −70 mV was chosen based on the steady-state inactivation curves as the membrane potential that does not lead to current activation (Figure 5) and that, however, produces little inactivation (Figure 6) in the absence of drugs. Upon depolarizing the membrane potential to −70 mV, from our standard holding potential of −110 mV, we quickly shift the equilibrium of VGSCs to pre-open–closed states (Armstrong, 2006). The equilibrium quickly favors pre-open–closed states over the resting states upon stepping the voltage to −70 mV since the voltage sensors of VGSCs move within a few milliseconds at −70 mV (Lacroix et al., 2013). In addition, a much slower process takes channels from those pre-open–closed states to an inactive state of the VGSCs (Figure 10) (Armstrong, 2006). Our rationale was that if EUG interacts with such pre-open–closed states, the available current to be activated by P_2_ in our custom protocol would decrease in amplitude with the extended duration of P_1_. We plotted the normalized average inhibition intensity of the total I_Na_ recorded with P_2_, induced by EUG, against P_1_ duration (Figure 10). For comparison, we repeated the same experiment and data analysis but using LID as the total I_Na_ inhibitor. Our data show that EUG, but not LID, inhibited I_Na_ after a conditioning pulse (P1) to −70 mV shorter than 100 ms in an exponential time course with a time constant of approximately 72 ms. For comparison, LID induces similar effects in decreasing the current at pulse P_2_ of this protocol, with kinetics of 709 ms. The kinetics of EUG inhibition potentiation by P_1_ was statistically different from that of LID (Student’s t-test, *p* = 0.04). In fact, at −70 mV, some level of inactivation exists (Figure 10A), but we cannot rule out the notion that EUG may bind to pre-open–closed states, and this effect would be reflected in the steady-state inactivation curves as well. This matter is currently being worked out in terms of a kinetic model to further strengthen this notion in a separate paper. There is currently limited information about drug binding to pre-open–closed states of mammalian VGSCs (Gilliam et al., 1989; Chernoff, 1990; Carmeliet and Mubagwa, 1998; Nesterenko et al., 2011), and this is one of our lines of investigation moving forward.

### The inhibitory mechanisms of EUG and LID on VGSCs are similar but not the same

**FIGURE 11:**
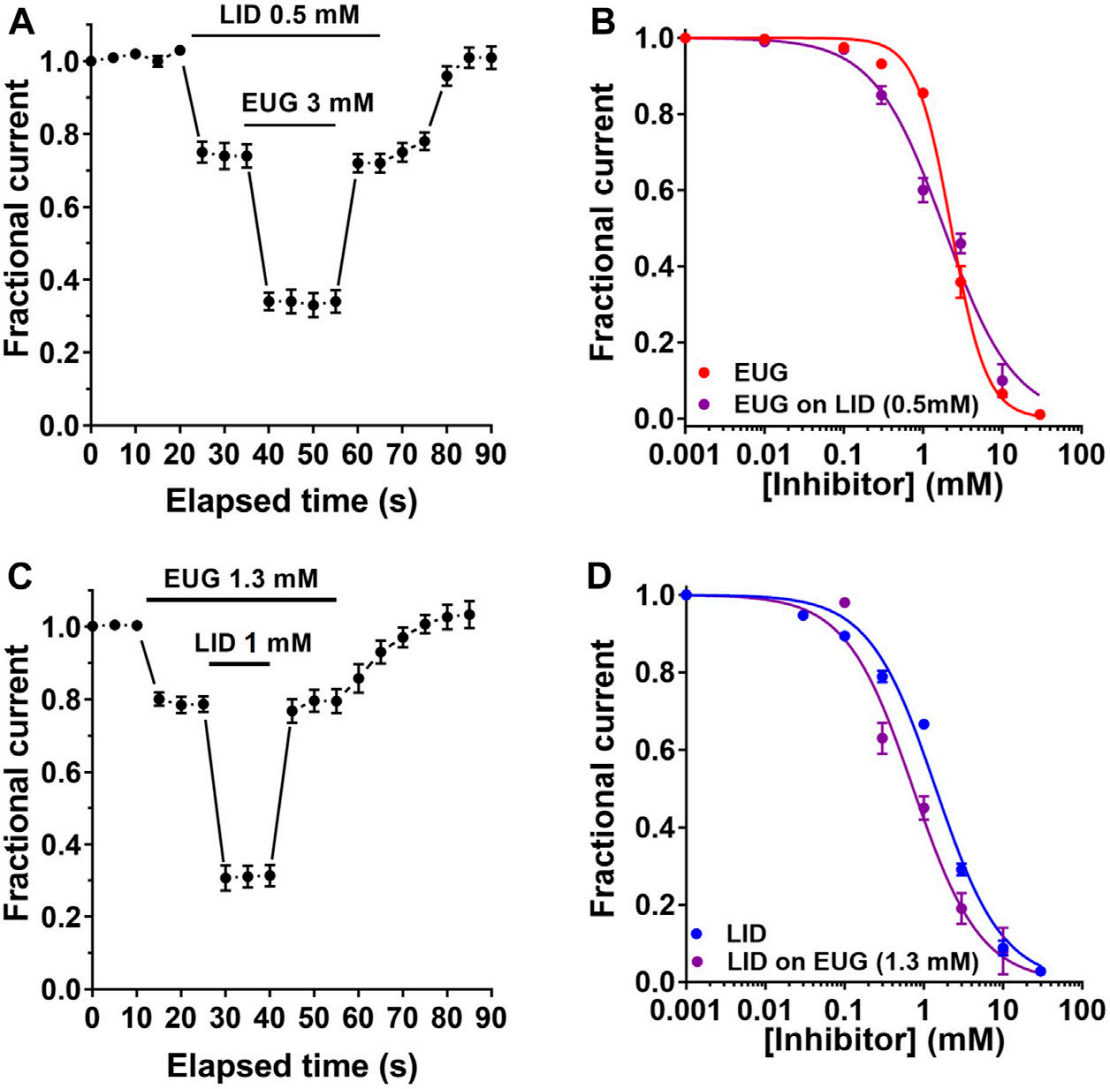
EUG and LID effects on VGSCs are interacting. **(A)** Time course of the double inhibition of the total I_Na_ by LID and EUG, as indicated. **(B)** After an approximate 25% I_Na_ pre-inhibition by LID at 0.5 mM, a dose–response curve for the inhibition by EUG was built. The IC_50_ value for EUG when inhibiting the total I_Na_ was 2.27 ± 0.07 mM. After a 25% pre-blockade by LID, EUG inhibits the total I_Na_ with a significantly lower IC_50_ of 1.84 ± 0.275 mM (extra sum-of-squares F-test, *p* < 0.01). The Hill coefficient of the EUG inhibition was also significantly reduced from 1.95 ± 0.120 without LID to 0.98 ± 0.140 after pre-inhibition with LID (extra sum-of-squares F-test, *p* < 0.0001). **(C)** Time course of the double inhibition of total I_Na_ by LID and EUG as indicated. **(D)** Similarly, after an approximate 25% I_Na_ pre-inhibition by EUG at 1.3 mM, a dose–response curve for the inhibition by LID was built. The IC_50_ value for LID when inhibiting the total I_Na_ was 1.42 ± 0.169 mM. After a 25% pre-inhibition by EUG, LID inhibits the total I_Na_ with a significantly lower IC_50_ value of 0.73 ± 0.126 mM (extra sum-of-squares F-test, *p* < 0.0001), with the Hill coefficient unchanged. Fractional inhibition data plotted against inhibitor concentrations were fitted using the Hill equation (Eq. 1, see text for details). Continuous lines plotted on b and d are best fits of the Hill equation to the color-coded data.

Our data suggest that EUG and LID may inhibit I_Na_ with a different mechanism. To further corroborate this notion, we built additional I_Na_-inhibiting dose–response curves by EUG and LID but in the presence of LID and EUG, respectively. First, we established a pre-inhibition of 25% of total I_Na_ with LID at 0.5 mM. This was planned to leave the other 75% of the current amplitude for the building of an inhibiting dose–response curve using EUG. All currents inhibited in these experiments were fully recovered upon drug washout. A ∼25% pre-inhibition of the total I_Na_ with LID was associated with a statistically significant change in the IC_50_ value for the inhibition by EUG. In the presence of LID at 0.5 mM, EUG inhibits the total I_Na_ with an IC_50_ value of 1.84 ± 0.274 mM (vs. 2.27 ± 0.075 mM in the absence of LID; extra sum-of-squares F-test, *p* < 0.01). In addition, the Hill coefficient for the inhibition of I_Na_ by EUG was reduced to 0.98 ± 0.140 mM (n = 12) by pre-inhibiting the total I_Na_ with LID (vs. 1.95 ± 0.120 with EUG alone; extra sum-of-squares F-test, *p* < 0.0001; Table 1; Figures 11A, B).

Next, we carried out the opposite experiment with regard to inhibitors. First, we pre-inhibited around 25% of the total I_Na_ with 1.3 mM EUG and proceeded with a dose–response curve for the inhibition of the remaining 75% of the currents with LID. This time, the remaining 75% of I_Na_ was inhibited by LID with a significantly lower IC_50_ value of 0.73 ± 0.126 mM (n = 10) vs. 1.42 ± 0.169 with LID alone (extra sum-of-squares F-test, *p* < 0.0001) and with no significant changes in the Hill coefficient compared with LID alone (Hill coefficient of 1) (Table 1; Figures 11C, D).

## Discussion

Here, we show, for the first time, a fast and fully reversible inhibition of VGSCs by EUG. The inhibition of I_Na_ (total and TTX-R) by EUG includes an interaction with the resting states of VGSCs with an affinity that is comparable but statistically different from the more potent inhibition produced by LID on the same currents (Figure 1; Table 1; dose–response curves compared by two-way ANOVA, *p* < 0.0001). Our data also suggest that EUG, different from LID, may interact with the pre-open–closed states of the VGSCs. Although speculative, this assumption is our best hypothesis to explain the hallmarks of the EUG inhibition of I_Na_: *i*) I_Na_ activation curves shift to more depolarized potentials (Figure 5); *ii*) steady-state inactivation curves shift to more negative potentials (Figure 6); *iii*) light changes in the recovery from fast inactivation (Figure 7); *iv*) light use-dependent inhibition of I_Na_ (Figure 8); and most importantly, *v*) the time-dependent enhanced inhibition of I_Na_ by a −70-mV membrane potential conditioning period (Figure 10).

EUG (1-allyl-4-hydroxy-3-methoxybenzene) is a naturally occurring compound present in the essential oil of many aromatic plants like clove, wormwood, and sweet basil. EUG has been used for a long time as a medical agent, food preservative, and flavoring agent. The excellent therapeutic index of EUG has been recently addressed in a comprehensive review that points out EUG as a generally acknowledged as safe (GRAS) chemical by the World Health Organization (WHO) (Tavvabi-Kashani et al., 2024). EUG is the ingredient responsible for analgesia in zinc eugenolate chelate, a dental cement material used in dentistry (FADM, 2001). EUG has been previously studied by many groups aiming at finding its ion channel-modulating properties, and it has been studied as a modulator of VGSCs (Park et al., 2006; Cho et al., 2008; Park et al., 2009; Moreira-Lobo et al., 2010), voltage-gated calcium channels (Sensch et al., 2000; Magyar et al., 2004; Lee et al., 2005; Chung et al., 2008; Seo et al., 2013), voltage-gated potassium channels (Erdélyi, 1999; Magyar et al., 2004; Li et al., 2007; Chung et al., 2008), GABAA channels, purinergic channels (Li et al., 2008), and TRP channels (Yang et al., 2003; Chung et al., 2014; Latorre and Baldion, 2020; Takahashi et al., 2021). These previous studies suggest EUG as a pore blocker of voltage-gated ion channels since no evidence has been found that can attest to any change in the biophysical properties of the channel voltage dependence or voltage sensitivity.

DRG neurons express several biophysically distinct VGSC isoforms, including Nav1.1, Nav1.6, Nav1.7, Nav1.8, and Nav1.9 (Berta et al., 2008; Ho and O’Leary, 2011; Shiers et al., 2020). Subunits Nav1.7, Nav1.8, and Nav1.9 are preferentially expressed in DRG neurons, and they are associated with mechanisms related to neuropathic and inflammatory pain (Chevrier et al., 2004; Maruyama et al., 2004; Liu and Wood, 2011; Bennett et al., 2019). Studies using VGSCs natively expressed in mammalian cells have the advantage of the channels being in their physiologic environment, a condition that is difficult to replicate when the channels are heterologously expressed in HEK cells, for instance. Studying VGSCs in their native membranes further increases the significance of the outcome data since only in that environment might the channels be inserted in their multiprotein complexes. Recent studies focus on ion channels and other membrane proteins as protein structures that are part of the multiprotein complexes in the membrane. Within these complexes, proteins are functionally coupled (Vacher et al., 2008; Abriel et al., 2015; Baronas et al., 2015; Subramanyam and Colecraft, 2015; Heijman and Dobrev, 2018). In the present study, we took advantage of those aspects in exchange for a more precise pharmacological study that a heterologous system of expression would provide in terms of studying an I_Na_ that is mediated by a single VGSC subtype.

### The affinity of I_Na_ inhibition by EUG

Previous studies have reported the inhibiting properties of EUG on VGSCs (Park et al., 2006; Cho et al., 2008; Park et al., 2009; Moreira-Lobo et al., 2010). In one of these studies, EUG was tested on the total I_Na_ and TTX-R I_Na_, both activated from a holding membrane potential of −80 mV (Cho et al., 2008). Their recordings show a considerable rundown of currents during the experiments. In addition, the inhibition of I_Na_ induced by EUG shown in that study was not fully recovered to its initial values. Finally, that study exposed the neurons to EUG for 10 min, which, together with the drug application technique, could have caused I_Na_ rundown during the course of the experiments. We propose that these four drawbacks contributed to a possible overestimation of EUG affinity to block I_Na_ in the study conducted by Cho et al. (2008).

Our dose–response curves were built with the fast inhibition of I_Na_ that was activated from a holding potential of −110 mV chosen to maximize VGSC recovery from inactivation, therefore I_Na_ maximization (Figures 1B–C). Our data demonstrate an immediate inhibition of the total I_Na_ or TTX-R I_Na_ by EUG or LID upon inhibitor perfusion onset, with 100% recovery upon inhibitor washout. Note that the I_Na_ amplitude of the currents we studied is stable throughout the time series, and they are rundown-free (Figure 1B). Our data shown in Figure 3 also demonstrate a rundown-free recording both under control conditions and in the presence of EUG or LID as I_Na_ inhibitors: when at maximal Na^+^ conductance activation at test potentials of +20 mV or more, all I-V curves are linear. Our approach was intended to estimate the affinity of the fast inhibition of I_Na_ by EUG. Our experiments using LID as the positive control serve as a validation for our method.

Our data show that EUG inhibits the total I_Na_ in DRG neurons with an IC_50_ value of 2.27 mM and a Hill coefficient of 1.95. EUG inhibits TTX-R I_Na_ with virtually the same properties, with an IC_50_ value of 2.21 mM and a Hill coefficient of 1.84 (Figure 1C; Table 1). The affinity of the binding of EUG to the resting states of VGSCs (by using a holding potential of −110 mV and a test pulse to +20 mV) was statistically different from the inhibition induced by LID, with an IC_50_ value of 1.42 mM, and TTX-R I_Na_ with an IC_50_ value of 0.44 mM, which agrees with previous reports (Kistner et al., 2010). EUG affinity to inhibit VGSCs is far enough for local anesthesia. For comparison, a popular 2% LID formulation for human local anesthesia is a solution of LID at 85 mM. Interestingly, the Hill coefficient of the dose responses when EUG is used to inhibit I_Na_, ∼2, is remarkably different from that when LID is used to inhibit the same currents (∼1). The Hill coefficient of dose–response curves is often related to binding sites, but the use of that knowledge is limited to orthosteric inhibitors such as pore blockers and not allosteric or state-dependent inhibitors (Bindslev, 2008; Prinz, 2010). Therefore, the only suggestion we can make, based on our data presented here and the Hill coefficients, is that EUG and LID may have different VGSC-inhibiting mechanisms. Additionally, in the case of a Hill coefficient of 2, for the inhibition of I_Na_ by EUG, we can rationalize that the coefficient does not arise from different EUG affinities to different VGSCs expressed in the neurons studied here. If that was the case, the slope of the dose–response curve would, instead, be shallower, thus showing an apparent Hill coefficient smaller than 1. Hence, we reinforce that since we deal with different VGSC isoforms here, with different kinetics and voltage dependencies, we simply speculate that the Hill coefficient we found for the I_Na_ inhibition by EUG, ∼2, indicates a different inhibiting mechanism compared to LID.

### EUG and LID may interact to inhibit I_Na_

Our data shown in Figure 11 suggest that EUG and LID may interact when blocking I_Na_. The 25% I_Na_ pre-inhibition with either drug induces a significant improvement on the inhibition by the other, which blocks the remaining 75% of the currents with a lower IC_50_ value compared to the drug alone (Table 1). In addition, pre-blocking I_Na_ with LID reduces the Hill coefficient of the EUG inhibition curve from ∼2 with EUG alone to ∼1 in the presence of LID. We are extremely cautious when interpreting these data, especially because they come from experiments with a natively expressed variety of different VGSCs that could be inhibited by EUG or LID with different mechanisms. Therefore, we took a very conservative approach and limited the conclusion of these experiments to report that EUG and LID inhibit I_Na_ with likely different mechanisms. New experiments using a single VGSC subunit expressed heterologously are necessary to continue the investigation of this aspect of I_Na_ inhibition by EUG and LID simultaneously.

### EUG and LID interact differently with distinct kinetic states of VGSCs

Our dose–response curves of inhibition of the total I_Na_ and TTX-R I_Na_ by EUG relate to the interaction of the inhibitor with the resting states of VGSCs. However, our detailed investigation revealed that EUG interacts with other states of VGSCs. Our data suggest that EUG may interact with VGSC pre-open–closed states possibly with a higher affinity than that of resting states (Figure 10). This notion is suggested by our experiments with a conditioning period at a membrane potential of −70 mV, which is known to populate pre-open–closed states of VGSCs (Armstrong, 2006), before I_Na_ is activated by a membrane potential step of +20 mV. Our data demonstrate that I_Na_ inhibition by EUG is intensified by such conditioning voltage steps, with a time constant of ∼70 ms, and we propose that this finding may be due to an interaction of EUG with the pre-open–closed states of VGSCs that are associated with a membrane potential of −70 mV. Specific experiments are still needed, but this piece of data alone suggests that such an interaction may take place with higher affinity than that with the resting states of the channels (when we activate the currents straight from a membrane potential of −110 mV). Our data using LID, an inhibitor known to bind to the inactivated states of VGSCs with higher affinity than to resting states, show the remarkable difference from that produced with EUG after time-varying −70-mV conditioning pulses. LID has its inhibiting effects on I_Na_ enhanced by the −70-mV conditioning pulse with a time constant of ∼700 ms. We propose that LID has its inhibiting potency increased in these experiments as more channels become inactivated, a slow process at −70-mV membrane potential (Armstrong, 2006). Differently, EUG would have its inhibiting potency increased faster (with a ∼70-ms time constant) as the pre-open–closed states, quickly achieved after stepping the membrane potential to −70 mV, are the states to which EUG would have an increased affinity. This notion will be a matter of further investigation in the near future using computation biology for kinetic models of this system.

We also propose that the right shift in the activation curves induced by EUG might be due to the interaction of EUG with pre-open–closed states of the VGSCs (Figure 5). Weak depolarizations would provide the channels with time to populate their pre-open–closed states, and that would be enough for EUG to further interact with the channels if the affinity to those states is higher than to resting states. LID does not show such a right shift in the voltage dependence of I_Na_ activation, in agreement with the notion that this drug would not interact with those pre-open–closed states. The remarkable effect of EUG on the steady-state inactivation curve of the total I_Na_ might also have been caused by the binding of EUG to pre-open–closed states. We propose the novel idea that a 100-ms conditioning pre-pulse, as shown in Figure 6A, is not a perfect measure of the binding of drugs to the fast inactivated states of VGSCs, even when we consider inactivation from pre-open–closed states. Other studies show that 1,000-ms or 10-s (10-fold or 100-fold longer than ours) conditioning pulses indeed show a remarkable shift in the V_*1/2*_ *-inact* of the available currents, as induced by LID (Clarkson et al., 1988; Sheets et al., 2010). In particular, with a 10-s conditioning pulse, LID shifted the available curve by 10 mV only. For comparison, EUG shifted the inactivation curve by 16 mV with a 100-ms conditioning pulse. Our 100-ms conditioning pre-pulses may not last enough to inactivate the channels and for an interaction with LID after that. Once again, LID, known to interact with the inactivated states of VGSCs, does not shift the inactivation curves with the intensity or with the short conditioning pulse as EUG does (Figure 6). We suggest that this voltage clamp protocol with a 100-ms conditioning pre-pulse may also evidence the drug binding to the pre-open–closed states of the channels, and we think that EUG induces a consistent 16-mV shift in these curves because it might interact with the pre-open–closed states of the channels during the conditioning pre-pulses around −70 mV.

In our view, protocols to test the recovery from inactivation at a −110-mV membrane potential (Figure 7), high frequencies of depolarizing time series (Figure 8), and rate of slow inactivation entry (Figure 9) are better ways to study the interaction of inhibitors with the inactivated states of VGSCs (Clarkson et al., 1988). We sustain this notion because in all these protocols, the membrane potential is held at −110 mV (the holding potential) or at +20 mV when I_Na_ are activated and inactivated. Neither of these voltages is associated with the pre-open–closed states of VGSCs (Lenaeus et al., 2017). EUG underperformed in all three voltage clamp protocols compared with LID. These results show that EUG is not an inhibitor that binds to the inactivated states of VGSCs, fast and slow, with a higher affinity than binds to the resting states of VGSCs.

Our data show that EUG does not possess the ability to interact with pre-open–closed states of TTX-R VGSCs. Therefore, this EUG property must be related to the TTX-S VGSCs that are expressed in DRG neurons, i.e., Nav1.1, Nav1.6, and Nav1.7. Interestingly, since we observed the state-dependent effects of EUG on the total I_Na_, i.e., on TTX-S plus TTX-R currents, we hypothesize that the observable effects on a TTX-S VGSC subunit would be even more clear.

EUG interacts with VGSCs, and this interaction modifies the voltage dependence of the channels. EUG may interact directly with the voltage sensors of the channels, as shown before for small molecules binding on extracellular residues of the homologous domain IV voltage sensor to cause a shift in the inactivation curve of the currents mediated by Nav1.3 (McCormack et al., 2013). EUD may also bind to the canonical local anesthetic binding site on S6 segments (the activation gate) that is allosterically connected to the voltage sensors of homologous domains III and IV (Sheets and Hanck, 2003; Sheets and Hanck, 2007). It is known that the activation of the voltage sensor of domain IV in VGSCs is necessary for the fast inactivation of these VGSCs (Kirsch et al., 1989; Campos et al., 2008). It seems that EUG does not interact with those regions since our data do not indicate stabilization of the inactivated states of VGSCs. Nevertheless, EUG may interact with pre-open–closed states of VGSCs as configured by the activation of voltage sensors of domains I and II or either one individually. Importantly, we cannot rule out that the voltage-dependent effect of EUG on the total I_Na_ reported here might be due to EUG interactions with an allosteric biding site elsewhere in the channel, including in the pore domain of the channels, a pathway we previously reported to be possible in Shaker K^+^ channels (Bassetto et al., 2021).

Another interesting and surprising piece of data in the present work is that EUG, but not LID, inhibits persistent total I_Na_ in DRG neurons (Figures 4A–D). It is important to mention that the inhibition of persistent I_Na_ could only be apparent if the inactivation kinetics of I_Na_ were accelerated. For example, we showed that neither EUG nor LID changed the kinetics of the inactivation process during a test pulse of the currents we studied (Figures 4E–H). Therefore, it is possible that EUG inhibits the specific channels that yield persistent currents more intensively than LID. This notion can lead to the inhibition of specific channels by EUG, and it will be investigated in our next studies. A previous study expressing the predominant cardiac VGSCs showed LID as an effective inhibitor of persistent voltage-activated Na^+^ currents (Dumaine and Kirsch, 1998). We propose that EUG might have a similar effect on the persistent I_Na_ observed in the present study.

## Conclusion

Our data show that EUG is a VGSC inhibitor with an affinity comparable with LID. The inhibiting mechanism of EUG on the resting states of the VGSCs overlaps with that of LID. EUG modifies the voltage-dependent activation and inactivation processes of I_Na_, and, different from LID, that effect might be due to the possible interaction of EUG with the pre-open–closed states of the VGSCs. Altogether, our data point out EUG as a state-dependent VGSC blocker different from LID, suggestively for those sensitive to TTX.

## Materials and methods

### General experimental procedures

All salts and drugs used in this work were purchased from Sigma (Sigma, St. Louis, MO). EUG was at least 98% pure, as stated by vendors. The pipette solution and the bath solutions were prepared directly from the salts and maintained at −20 °C until the day of the experiment, when they were thawed. A solution of eugenol (stock solution) was also prepared in advance to a concentration of 1 M in ethanol and stored at −20 °C. Just before the experiment, the stock solution was diluted in the bath solution to the desired final concentration of eugenol and sonicated for 5 min. The maximal final concentration of ethanol used in this study was 0.73% vol/vol, which, according to our own data (Figure 2) and data obtained by others (Wu and Kendig, 1998; Horishita and Harris, 2008), inhibits I_Na_ in approximately 10% and without any change in the voltage dependence of the activation and inactivation processes. This solution containing eugenol was applied over a single cell through a home-made pressurized perfusion system connected to a double-barreled capillary (Harvard Apparatus, Holliston, MA) to deliver the solutions with and without eugenol in the vicinity of the tested cell, with very fast interchangeable capability.

### Cell preparation

DRG neurons from 1–3-day-old rats were used as a model of natively expressed VGSCs. The cell preparation procedure was adapted from the study by Kostyuk et al. (1981). In brief, the rats were euthanized by decapitation, the vertebral canal was opened, and DRGs were quickly removed. All animals were handled in compliance with the Guide for the Care and Use of Laboratory Animals by the US National Institutes of Health (NIH Publication 85–23, revised 1996; http://www.nap.edu/.readingroom/books/labrats/index.html). In addition, all experimental protocols were approved by the Comissão de Ética no Uso de Animais (CEUA), a collegiate body linked to the Congregation of the Institute of Biomedical Sciences of the University of São Paulo (ICB-USP). Ganglia were digested with trypsin 0.25% in a Ca^2+^, Mg^2+^-free Earle´s balanced salt solution (EBSS) containing (mM) 132.8 NaCl, 5.3 KCl, 1 NaH_2_PO_4_, 5.5 glucose, and 10 HEPES, pH 7.4. After digestion, the ganglia were mechanically reduced using a fire-polished Pasteur pipette in a Ca^2+^, Mg^2+^-free EBSS containing 5 U/mL DNAse (type I; Sigma) and 0.15% trypsin inhibitor (type IS; Sigma), supplemented with 10% fetal calf serum. After pelleting, the cells were resuspended in Dulbecco's modified Eagle's medium (DMEM; Sigma) supplemented with 10% fetal calf serum, 100 UI/mL penicillin, and 100 mg/mL streptomycin and seeded on glass coverslips previously treated with poly-L-lysine. The cells were kept in a water-jacket incubator at 37 °C and a 5% CO_2_ atmosphere until just before experiments, which were carried out during the first 7 days after cell isolation.

### Electrophysiology

Voltage-activated Na^+^ currents passing through VGSCs were recorded using voltage clamping with the conventional whole-cell patch-clamp configuration (Hamill et al., 1981). Patch electrodes were fabricated from borosilicate glass capillaries using a model PB-7 micropipette puller (Narishige, Tokyo, Japan). Recording pipettes were pulled from borosilicate glass to achieve initial bath resistances averaging 2 MΩ and were filled with an intracellular solution containing (mM) 10 NaCl, 150 CsF, 10 TEA chloride, 1 ATP, 4.5 MgCl_2_, 9 EGTA, and 10 HEPES, pH 7.3. CsCl and TEA chloride were used in order to eliminate K^+^ currents. Cells were bathed during the recordings in an extracellular solution containing (mM) 82 choline chloride, 50 NaCl, 1.2 MgCl_2_, 1.8 CaCl_2_, 1 CoCl_2_, 4 KCl, 5 glucose, and 10 HEPES, pH 7.4. Na^+^ was partially replaced by choline (82 mM) to decrease the sodium driving force, avoid overload of the amplifier, and ensure a good voltage clamp. Co^2+^ in the extracellular solution was used to eliminate Ca^2+^ currents. After achieving a high-resistance seal, the whole-cell configuration was usually established by applying negative pressure to the pipette. Typical access resistance values were below 5 MΩ. Command voltage waveforms were generated in a computer using Clampex 10 software (Molecular Devices, Foster City, CA), and a DAC interface (model 1322; Molecular Devices, Foster City, CA) delivered the analog voltage signal to the Axopatch 200B patch-clamp amplifier (Molecular Devices, Sunnyvale, CA), which maintained the pipette voltage. The P/-4 protocol was used to remove voltage-independent leak currents and uncompensated capacitance (Bezanilla and Armstrong, 1977). The current recordings were low-pass filtered at 2 kHz by a built-in Bessel filter in the amplifier, sampled at 25 kHz in 16-bit digital levels by an ADC, and recorded on a hard disk for ulterior analysis. The cell membrane capacitance was canceled, and access resistance was routinely compensated (85% for both prediction and compensation; lag set to 10 ms). The holding potential in all experiments was set to −110 mV to avoid inactivation of the VGSC. All recordings were performed between 20°C and 23 °C. The recording chamber was continuously perfused with the bath solution to avoid unstirred layers. Recording cells exhibiting an I_Na_ peak of less than 500 pA throughout several depolarizing voltages were discarded. AP were recorded using the same cell preparation but in the current-clamp mode and using a pipette solution containing (mM) 10 NaCl, 150 KCl, 1 ATP, 4.5 MgCl_2_, 9 EGTA, and 10 HEPES, pH 7.3, and a bath solution, including the solution where eugenol was dissolved, containing (mM) 132 NaCl, 1.2 MgCl_2_, 1.8 CaCl_2_, 4 KCl, 5 glucose, and 10 HEPES, pH 7.4.

### Data analysis and graphs

Scientific data were processed, analyzed, and plotted using Clampfit (Molecular Devices, Foster City, CA), GraphPad Prism (GraphPad Software, LLC, La Jolla, CA), Origin (OriginLab, Northampton, MA), and Microsoft Excel (Microsoft, Redmond, WA). All the graphs represent mean values from at least six independent experiments, unless otherwise noted. The vertical bars in the graphs are the standard error of the mean (SEM).

The fitting functions mentioned in the *Results* section are as follows:

Dose–response curves were fitted with the Hill formalism:

$$Normalized\;\left(uninhibited\right)\;current=\frac{{\left[EUG\right]}^{nH}}{{\left[EUG\right]}^{nH}+{{IC}_{50}}^{nH}},$$ (1)

where the *Normalized (uninhibited) current* is the remaining I_Na_ after inhibition by EUG or LID (the Fractional current in the graphs), *IC*_*50*_ is the concentration of eugenol that inhibits 50% of the I_Na_, and *nH* is the Hill coefficient.

I_Na_ inactivation kinetics were fitted by the following exponentials:

$$I_{Na}=\left(A_{1}\times 1-e^{-t/\tau_{1}}\right)+\left(A_{2}\times {1-e}^{-t/\tau_{2}}\right)+y_{0},$$ (2)

where *I*_*Na*_ is the Na^+^ current at a given moment *t*, *A*_*1-2*_ are the weights of the respective exponentials, *τ*_*1-2*_ are their time constants, and *y*_*0*_ is an adjusting factor for persistent currents. Inactivation time constants were calculated as follows:

$$Inactivation\;time\;constant=\frac{\left(\left(A_{1}\times \tau_{1}\right)+\left(A_{2}\times \tau_{2}\right)\right)}{\left(\left(A_{1}+A_{2}\right)\right)}.$$ (3)

Current–voltage relationships were transformed into Na^+^ conductance activation by voltage (G-V) curves by using Ohm’s law:

$$G_{m}=\frac{I_{peak}}{V_{m}-V_{r}},$$ (4)

where *G*_*m*_ is the equivalent conductance at *Ipeak*, which, in turn, is the current peak value at the given voltage, *V*_*m*_ is the membrane potential, and *V*_*r*_ is the reversal potential for Na^+^.

Na^+^ conductance activation by voltage (G-V) curves was fitted by the following equation:

$$Normalized\;{Na}^{+}conductance=\frac{1}{1+e^{\left(\frac{V_{1/2-act}-V_{m}}{\mathrm{Max}\;slope-act}\right)}},$$ (5)

where *Normalized Na* ^*+*^ *conductance* is the fractional conductance activated at a given membrane potential *V*_*m*_, *V*_*1/2-act*_ is the membrane potential for half-maximal Na^+^ conductance activation (the midpoint), and *Max slope-act* is the voltage sensitivity of the activation by voltage process.

I_Na_ inactivation by voltage curves (inactivation curves) was fitted by the following equation:

$$Available\;{Na}^{+}\;current=1-\frac{1}{1+e^{\left(\frac{V_{\frac{1}{2}-inact}-V_{m}p-p}{\mathrm{Max}\;slope-inact}\right)}},$$ (6)

where *Available Na* ^*+*^ *current* is the Na^+^ current after the conditioning pre-pulse voltage period *V*_*m*_ *p-p*, *V*_*1/2-inact*_ is the *V*_*m*_ *p-p* that inactivates half of I_Na_, and *Max slope-inact* is the voltage sensitivity of the inactivation by voltage process.

The kinetics of I_Na_ recovery from inactivated states were fitted with the following sum of exponentials:

$$\frac{P_{3}}{P_{1}}current\;ratio=\left(\frac{\%fast}{100}\times {1-e}^{\frac{{-P}_{2}duration}{\tau_{fast}}}\right)+\left(\left(1-\frac{\%fast}{100}\right)\times {1-e}^{\frac{-P_{2}duration}{\tau_{slow}}}\right),$$ (7)

where *P*_*3*_ */P*_*1*_ *current ratio* is the fractional I_Na_ recovered from inactivation during *P*_*2*_, *%fast* is the % fraction of the fast recovery from inactivation component amplitude, *τ*_*1*_ is the faster time constant of the I_Na_ recovery from inactivation, and *τ*_*2*_ is the slower time constant of the I_Na_ recovery from inactivation.

$$\frac{P_{3}}{P_{1}}current\;ratio=Plateau+\left(\left(Y_{P1=2ms}-Plateau\right)\times \frac{\%fast}{100}\times e^{-P_{1}duration/\tau_{fast}}\right)+\left(\left(Y_{P1=2ms}-Plateau\right)\times \left(1-\frac{\%fast}{100}\right)\times e^{-P_{1}duration/\tau_{slow}}\right),$$ (8)

where *P*_*3*_ */P*_*1*_ *current ratio* is the fractional I_Na_ inactivated during *P*_*1*_ and not recovered from inactivated during *P*_*2*_ (see text for details), *Plateau* is the current level at P_1_>>10000 ms, *Y*_*P1=2ms*_ is the *P*_*3*_ */P*_*1*_ current ratio when P_1_ = 2 ms, *%fast* is the % fraction of the fast inactivation component, *τ*_*1*_ is the faster time constant of the slow inactivation component, and *τ*_*2*_ is the slower time constant of the slow inactivation component.

### Statistical analysis

Data from individual cells were treated individually, including for fitting purposes. Pooled fitting parameters from different groups, e.g., EUG vs. control (its absence), were compared using a paired *t*-test to detect consistent changes in the parameters that relate to the drugs. Levels of significance were *p < 0.05, **p < 0.01, ***p < 0.001, and ****p < 0.0001.

## Summary

Eugenol is an aromatic substance obtained from the essential oil of many plants that produces analgesia by a still uncertain mechanism. Here, we show comprehensive data indicating that eugenol inhibits voltage-gated Na^+^ channels with a mechanism that is different from lidocaine. We propose, based on the interpretation of our findings, that eugenol inhibits voltage-gated Na^+^ channels by interacting with their resting, pre-open–closed, and inactivated states.

## Data Availability

The original contributions presented in the study are included in the article/Supplementary Material; further inquiries can be directed to the corresponding author.
