# Supplementary material for: Eugenol and lidocaine inhibit voltage-gated Na+ channels from dorsal root ganglion neurons with different mechanisms
Source: Front Pharmacol. 2024 Jun 26;15:1354737. doi: 10.3389/fphar.2024.1354737 (PMC11234063; doi:10.3389/fphar.2024.1354737)
Supplement: Supplementary file 1 [file DataSheet1.PDF]

Eugenol and lidocaine may have different binding sites on voltage gated Na^+ channels

Luiz Moreira-Junior[1], Jose Henrique Leal-Cardoso[2], Antonio Carlos Cassola[3], and Joao Luis Carvalho-de-Souza[1,3,#]

[1]Department of Anesthesiology, University of Arizona, Tucson, AZ 85724
[2]Superior Institute of Biomedical Sciences, State University of Ceará, Campus of Itaperi, Fortaleza, CE, Brazil 607402
[3]Department of Physiology and Biophysics, Biomedical Sciences Institute, University of Sao Paulo, São Paulo, SP, Brazil 05508

[#]Present address: Department of Anesthesiology, University of Arizona, Tucson, AZ 85724

Supplementary information

Table S1.1. Dose response curves' data.

Conc.	EUG			LID			EUG (LID 0.5 mM)			LID (EUG 1.3 mM)			MEUG		
(mM)	Average	SEM	n	Average	SEM	n	Average	SEM	n	Average	SEM	n	Average	SEM	n
**0.001**	1.00	0.000	15	1.00	0.000	21				1.00	0.000	6			
**0.01**	1.00	0.002	14				0.99	0.001	6						
**0.03**				0.95	0.006	23									
**0.05**															
**0.1**	0.98	0.003	15	0.89	0.010	24	0.97	0.008	6	0.98	0.010	7	0.99	0.006	10
**0.15**															
**0.3**	0.93	0.006	13	0.79	0.015	19	0.85	0.023	7	0.63	0.040	6			
**1**	0.86	0.008	12	0.67	0.011	28	0.60	0.032	8	0.45	0.030	7	0.93	0.004	15
**1.5**															
**1.75**															
**2**															
**3**	0.36	0.042	15	0.29	0.015	20	0.46	0.026	6	0.19	0.040	6	0.83	0.020	18
**4**															
**5**													0.86	0.010	16
**7**															
**10**	0.07	0.013	13	0.09	0.019	14	0.10	0.043	6	0.08	0.060	6	0.31	0.024	15
**15**													0.14	0.026	12
**20**													0.00	0.008	11
**30**	0.01	0.011	14	0.03	0.013	21									

Table S1.2. Dose response curves' data.

Conc.	EST			AEUG			GUA			ZIN			VAN		
(mM)	Average	SEM	n	Average	SEM	n	Average	SEM	n	Average	SEM	n	Average	SEM	n
0.001															
0.01															
0.03															
0.05															
0.1				0.97	0.003	31									
0.15															
0.3	1.00	0.000	7												
1				0.84	0.006	23	0.95	0.011	10	0.91	0.004	37	0.96	0.003	22
1.5															
1.75															
2	0.99	0.006	14	0.86	0.007	22	0.87	0.010	19						
3	0.86	0.015	16												
4	0.22	0.021	14												
5	0.27	0.036	18	0.54	0.015	25	0.76	0.015	13	0.62	0.027	25	0.70	0.020	21
7	0.08	0.016	10												
10	0.02	0.012	6	0.10	0.012	19	0.52	0.036	10	0.24	0.015	25	0.45	0.012	32
15															
20							0.54	0.029	13	0.03	0.005	18	0.06	0.012	18
30															

Table S1.3. Dose response curves' data.

Conc.	IEUG			ANE			EUG (TTX-R)			LID (TTX-R)		
(mM)	Average	SEM	n	Average	SEM	n	Average	SEM	n	Average	SEM	n
**0.001**	1.00	0.000	9				1.00	0.000	6	1.00	0.000	11
**0.01**	0.99	0.003	23				0.99	0.002	25	0.82	0.010	14
**0.03**												
**0.05**	0.86	0.007	24									
**0.1**	0.78	0.013	21				0.96	0.003	17	0.78	0.012	23
**0.15**	0.50	0.020	29									
**0.3**							0.92	0.004	20	0.55	0.010	24
**1**				0.98	0.004	28	0.85	0.017	26	0.38	0.017	24
**1.5**				0.97	0.003	28						
**1.75**				0.70	0.018	32						
**2**				0.18	0.020	15						
**3**	0.40	0.015	11	0.21	0.022	22	0.34	0.030	24	0.26	0.017	19
**4**												
**5**				0.02	0.007	8						
**7**												
**10**	0.02	0.008	19	0.03	0.009	14	0.14	0.040	13	0.06	0.013	17
**15**												
**20**	0.00	0.002	9									
**30**							0.00	0.000	13	0.00	0.000	10

Table S2. Time series of EUG and LID's total and TTX-R I_{Na} blockade.

Time	Condition	Total I_{Na}						TTX-R I_{Na}					
		EUG			LID			EUG			LID		
(sec)		Average	SEM	n	Average	SEM	n	Average	SEM	n	Average	SEM	n
0	Control (baseline)	1.00	0.000	25	1.00	0.000	23	1.00	0.000	25	1.000	0.000	15
5		1.00	0.003	25	1.00	0.003	23	1.00	0.006	25	0.997	0.011	15
10		1.00	0.004	25	1.01	0.003	23	1.00	0.007	25	1.020	0.007	15
15	Drug exposure	0.55	0.020	25	0.63	0.018	23	0.62	0.020	25	0.700	0.018	15
20		0.54	0.021	25	0.63	0.018	23	0.62	0.018	25	0.688	0.020	15
25		0.54	0.021	25	0.63	0.018	23	0.63	0.018	25	0.698	0.019	15
30	Recovery	0.96	0.017	25	0.99	0.012	23	1.00	0.017	25	0.954	0.017	15
35		0.99	0.015	25	1.01	0.010	23	1.04	0.019	25	0.981	0.014	15
40		0.99	0.012	25	1.01	0.010	23	1.03	0.017	25	1.000	0.016	15

Table S3. Membrabne resistance (MΩ) of individual cells before and after exposure to EUG 3 mM.

Cell	Before (control)	EUG
**1**	145.49	148.9
**2**	169.51	171.41
**3**	181.14	172.79
**4**	181.88	163.83
**5**	637.09	828.28
**6**	716.16	690.18
**7**	178.91	188.31
**8**	188.24	188.3
**9**	155.94	155.05
**10**	137.77	137.89
**11**	368.75	372.68
**12**	107.2	102.77
**13**	93.34	96.73
**14**	394.52	335.87
**15**	459.34	478.4
**16**	414.13	443.12
**17**	647.33	619.22
**18**	137.21	153.08
**19**	84.59	87.91

Table S4. Membrane potential for half-maximal Na^+ conductance activation ($V_{1/2\text{-}act}$, mV) in individual cells, before and after exposure to EUG 2 mM.

Cell*	Total I_{Na}		TTX-R I_{Na}	
	Before (control)	EUG	Before (control)	EUG
1	-19.70	-10.73	-8.899	-9.916
2	-10.65	-7.173	-16.79	-13.33
3	-29.98	-19.64	-12.05	-16.67
4	-12.99	-5.137	-7.394	-10.24
5	-8.352	-8.98	-8.603	-5.511
6	-28.69	-24.01	-10.13	-5.684
7	-16.95	-14.91	-21.13	-19.13
8	-24.71	-13.00		
9	-26.76	-20.84		

*The cell number does mean same cell in Total and in TTX-R I_{Na} experiments.

Table S5. Maximal voltage sensitivity of the activation by voltage process (Max *slope-act*, mV/efold) in individual cells, before and after exposure to EUG 2 mM.

Cell*	Total I_{Na}		TTX-R I_{Na}	
	Before (control)	EUG	Before (control)	EUG
1	3.614	5.526	6.326	6.818
2	3.179	5.494	4.157	6.311
3	2.637	4.967	5.682	5.452
4	4.015	6.616	4.001	4.894
5	3.634	5.996	7.838	8.267
6	2.556	6.156	5.745	6.510
7	2.533	4.306	5.977	6.698
8	1.954	2.717		
9	4.339	6.496		

*The cell number does mean same cell in Total and in TTX-R I_{Na} experiments.

Table S6. Membrane potential for half-maximal Na^+ conductance activation ($V_{1/2\text{-}act}$, mV) in individual cells, before and after exposure to LID 1 mM.

Cell*	Total I_{Na}		TTX-R I_{Na}	
	Before (control)	LID	Before (control)	LID
1	-13.9	-10.84	-27.07	-25.02
2	-18.36	-17.68	-22.73	-21.81
3	-16.99	-19.58	-24.62	-25.87
4	-11.19	-11.29	-20.94	-25.3
5	-19.55	-18.64	-3.298	2.959
6			-6.882	-9.166
7			-8.03	-6.571
8			-23.74	-19.84
9			-8.469	-6.716

*The cell number does mean same cell in Total and in TTX-R I_{Na} experiments.

Table S7. Maximal voltage sensitivity of the activation by voltage process (Max *slope-act*, mV/efold) in individual cells, before and after exposure to LID 1 mM.

Cell*	Total I_{Na}		TTX-R I_{Na}	
	Before (control)	LID	Before (control)	LID
1	2.814	3.305	2.15	2.416
2	4.717	5.718	3.585	6.095
3	4.137	5.241	6.54	7.402
4	5.644	6.129	6.413	6.184
5	6.02	6.443	7.164	9.974
6			6.625	8.405
7			8.865	10.98
8			6.308	7.92
9			7.274	8.84

*The cell number does mean same cell in Total and in TTX-R I_{Na} experiments.

Table S8. Membrane potential for half-maximal Na^+ conductance inactivation ($V_{1/2\text{-}inact}$, mV) in individual cells, before and after exposure to EUG 2 mM.

Cell*	Total I_{Na}		TTX-R I_{Na}	
	Before (control)	EUG	Before (control)	EUG
1	-52.46	-63.75	-24.64	-28.59
2	-41.4	-57.14	-30.99	-33.94
3	-57.89	-69.63	-27.12	-32.48
4	-37.54	-55.48	-28.96	-34.07
5	-54.62	-75.79	-31.87	-46.4
6	-40.66	-59.29	-43.81	-44.79
7	-51.91	-57.64	-34.62	-37.94
8	-54.6	-78.67		
9	-44.97	-57.24		

*The cell number does mean same cell in Total and in TTX-R I_{Na} experiments.

Table S9. Maximal voltage sensitivity of the inactivation by voltage process (Max *slope-inact*, mV/efold) in individual cells, before and after exposure to EUG 2 mM.

Cell*	Total I_{Na}		TTX-R I_{Na}	
	Before (control)	EUG	Before (control)	EUG
1	-13.38	-14.8	-5.123	-5.184
2	-11.97	-10.65	-7.713	-6.728
3	-13.1	-13.47	-5.831	-5.779
4	-7.857	-10.21	-3.583	-3.945
5	-11.13	-17.93	-6.674	-7.158
6	-8.324	-9.046	-5.373	-6.792
7	-8.291	-7.985	-5.572	-5.233
8	-10.83	-14.87		
9	-9.654	-11.22		

*The cell number does mean same cell in Total and in TTX-R I_{Na} experiments.

Table S10. Membrane potential for half-maximal Na^+ conductance inactivation ($V_{1/2\text{-}inact}$, mV) in individual cells, before and after exposure to LID 1 mM.

Cell*	Total I_{Na}		TTX-R I_{Na}	
	Before (control)	LID	Before (control)	LID
1	-38.46	-42.62	-19.87	-29.54
2	-52.87	-59.61	-25.79	-30.3
3	-48.71	-54.7	-31.69	-34.82
4	-40.89	-48.47	-29.13	-34
5	-53.16	-56.32	-21.06	-13.3
6			-26.27	-32.45
7			-37.36	-43.07
8			-37.41	-41.77
9			-30.45	-33.88

*The cell number does mean same cell in Total and in TTX-R I_{Na} experiments.

Table S11. Maximal voltage sensitivity of the inactivation by voltage process (Max *slope-inact*, mV/efold) in individual cells, before and after exposure to LID 1 mM.

Cell*	Total I_{Na}		TTX-R I_{Na}	
	Before (control)	LID	Before (control)	LID
1	-9.114	-10.01	-5.1	-4.454
2	-9.662	-11.35	-5.191	-5.078
3	-8.614	-9.163	-4.307	-4.311
4	-12.09	-11.45	-4.777	-4.855
5	-12.93	-10.7	-6.296	-12.65
6			-5.162	-5.321
7			-8.809	-10.59
8			-7.236	-13.14
9			-4.974	-5.017

*The cell number does mean same cell in Total and in TTX-R I_{Na} experiments.

Table S12. Fast component ration of the I_{Na} recovery from inactivation in individual cells, before and after exposure to EUG 2 mM.

Cell*	Total I_{Na}		TTX-R I_{Na}	
	Before (control)	EUG	Before (control)	EUG
1	86.94	78.09	78.52	84.44
2	82.73	70.94	80.63	78.86
3	76.69	75.58	74.57	46.14
4	78.96	76.09	72.65	66.84
5	83.38	78.3	88.78	82.74
6	78.15	76.52		
7	72.49	61.13		

*The cell number does mean same cell in Total and in TTX-R I_{Na} experiments.

Table S13. Fast and slow time constants of the I_{Na} recovery from inactivation in individual cells, before and after exposure to EUG 2 mM.

Cell*	Total I_{Na}				TTX-R I_{Na}			
	Fast component		Slow component		Fast component		Slow component	
	Before (control)	EUG	Before (control)	EUG	Before (control)	EUG	Before (control)	EUG
1	13.86	13.17	165.1	176.30	3.23	1.68	118.1	247.7
2	9.95	8.40	96.37	143.90	2.26	1.62	165.2	243.5
3	7.03	6.03	53.80	107.20	5.38	6.81	176.8	142.3
4	6.48	9.66	58.11	176.50	2.93	2.02	169.6	269.2
5	7.50	9.41	71.80	192.50	2.55	1.70	91.94	80.62
6	14.22	17.13	88.66	235.60				
7	8.33	7.75	89.50	95.33				

*The cell number does mean same cell in Total and in TTX-R I_{Na} experiments.

Table S14. Fast component ration of the I_{Na} recovery from inactivation in individual cells, before and after exposure to LID 1 mM.

Cell*	Total I_{Na}		TTX-R I_{Na}	
	Before (control)	LID	Before (control)	LID
1	86.65	21.28	80.13	59.29
2	52.46	21.12	68.11	52.71
3	57.65	6.952	78.05	61.22
4	42.99	8.821	55.38	61.33
5	77.13	36.58	79.2	47.45
6	62.17	34.56		
7	77.3	39.65		

*The cell number does mean same cell in Total and in TTX-R I_{Na} experiments.

Table S15. Fast and slow time constants of the I_{Na} recovery from inactivation in individual cells, before and after exposure to LID 1 mM.

Cell*	Total I_{Na}				TTX-R I_{Na}			
	Fast component		Slow component		Fast component		Slow component	
	Before (control)	LID	Before (control)	LID	Before (control)	LID	Before (control)	LID
**1**	6.97	19.50	48.40	171.70	2.19	4.00	208.07	301.57
**2**	19.01	50.28	214.31	327.44	1.19	5.14	23.93	116.65
**3**	13.93	17.38	46.93	105.00	1.73	4.43	7.28	332.45
**4**	10.37	26.96	46.42	119.05	0.90	4.94	3.27	805.15
**5**	15.16	50.81	77.46	392.31	1.86	2.24	301.48	236.57
**6**	15.92	33.84	73.47	379.79				
**7**	17.19	24.9	102.06	275.10				

*The cell number does mean same cell in Total and in TTX-R I_{Na} experiments.

Table S16. Frequency dependent blockade in individual cells. Data are shows as fractional current after blockade by EUG 2 mM, in the first pulse (no frequency dependent blockade effect) and in the 20th pulse (frequancy dependent blockade added)..

Cell*	Total I_{Na}				TTX-R I_{Na}			
	2 Hz		5 Hz		2 Hz		5 Hz	
	1st pulse	20th pulse	1st pulse	20th pulse	1st pulse	20th pulse	1st pulse	20th pulse
**1**	0.40	0.36	0.4	0.31	0.62	0.56	0.75	0.66
**2**	0.52	0.54	0.56	0.47	0.6	0.48	0.63	0.54
**3**	0.58	0.56	0.58	0.54	0.47	0.36	0.55	0.36
**4**	0.37	0.34	0.35	0.31	0.55	0.46	0.51	0.43
**5**	0.28	0.15	0.38	0.12			0.55	0.38
**6**	0.50	0.49	0.52	0.46				
**7**	0.730	0.74	0.28	0.17				
**8**	0.29	0.26	0.63	0.55				
**9**	0.53	0.56						

*The cell number does mean same cell in Total and in TTX-R I_{Na} experiments.

Table S17. Frequency dependent blockade in individual cells. Data are shows as fractional current after blockade by LID 1 mM, in the first pulse (no frequency dependent blockade effect) and in the 20th pulse (frequancy dependent blockade added).

Cell*	Total I_{Na}				TTX-R I_{Na}			
	2 Hz		5 Hz		2 Hz		5 Hz	
	1st pulse	20th pulse	1st pulse	20th pulse	1st pulse	20th pulse	1st pulse	20th pulse
1	0.42	0.31	0.73	0.53	0.58	0.3	0.55	0.18
2	0.6	0.51	0.48	0.06	0.49	0.3	0.51	0.15
3	0.65	0.57	0.51	0.07	0.64	0.5	0.68	0.34
4	0.46	0.34	0.60	0.25	0.53	0.51	0.68	0.50
5	0.55	0.45	0.51	0.26	0.56	0.46	0.62	0.42
6	0.48	0.42			0.59	0.57	0.69	0.0
7					0.66	0.58	0.67	0.50
8							0.68	0.41

*The cell number does mean same cell in Total and in TTX-R I_{Na} experiments.

Table S18. Slow inactivation stateentry kinetics of I_{Na} before and during exposure to EUG 2 mM or LID 1 mM.

Cell*	Total I_{Na}				TTX-R I_{Na}			
	EUG		LID		EUG		LID	
	Before (control)	EUG	Before (control)	LID	Before (control)	EUG	Before (control)	LID
1	803.20	713.90	329.10	84.13	259.90	167.30	360.80	210.90
2	4145.00	1928.00	128.40	39.12	818.30	405.40	510.80	156.80
3	748.10	344.50	72.72	7.878	1477.00	711.30	371.90	90.580
4	5328.00	1032.00	199.40	39.45	338.30	255.80	397.10	123.40
5	812.00	1066.00	2740.00	54.65	1070.00	558.80	276.90	113.30
6			416.40	64.16			265.30	141.10
7			671.60	93.23				
			1549.00	93.13				
			1303.00	23.05				
			663.90	38.88				
			765.30	25.47				

*The cell number does mean same cell in Total and in TTX-R I_{Na} experiments.
